# The Assessment, Management and Prevention of Calf Muscle Strain Injuries: A Qualitative Study of the Practices and Perspectives of 20 Expert Sports Clinicians

**DOI:** 10.1186/s40798-021-00364-0

**Published:** 2022-01-15

**Authors:** Brady Green, Jodie A. McClelland, Adam I. Semciw, Anthony G. Schache, Alan McCall, Tania Pizzari

**Affiliations:** 1 La Trobe Sport and Exercise Medicine Research Centre, La Trobe University, Melbourne, Australia; 2 Northern Centre for Health Education and Research, Northern Health, Epping, VIC Australia; 3 Arsenal Performance and Research Team, Arsenal Football Club, London, UK; 4 School of Applied Sciences, Edinburgh-Napier University, Edinburgh, UK

**Keywords:** Calf, Soleus, Gastrocnemius, Injury, Return to play, Rehabilitation, Recurrence

## Abstract

**Background:**

Despite calf muscle strain injuries (CMSI) being problematic in many sports, there is a dearth of research to guide clinicians dealing with these injuries. The aim of this study was to evaluate the current practices and perspectives of a select group of international experts regarding the assessment, management and prevention of CMSI using in-depth semi-structured interviews.

**Results:**

Twenty expert clinicians working in elite sport and/or clinician-researchers specialising in the field completed interviews. A number of key points emerged from the interviews. Characteristics of CMSI were considered unique compared to other muscle strains. Rigor in the clinical approach clarifies the diagnosis, whereas ongoing monitoring of calf capacity and responses to loading exposure provides the most accurate estimate of prognosis. Athlete intrinsic characteristics, injury factors and sport demands shaped rehabilitation across six management phases, which were guided by key principles to optimise performance at return to play (RTP) while avoiding subsequent injury or recurrence. To prevent CMSI, periodic monitoring is common, but practices vary and data are collected to inform load-management and exercise selection rather than predict future CMSI. A universal injury prevention program for CMSI may not exist. Instead, individualised strategies should reflect athlete intrinsic characteristics and sport demands.

**Conclusions:**

Information provided by experts enabled a recommended approach to clinically evaluate CMSI to be outlined, highlighting the injury characteristics considered most important for diagnosis and prognosis. Principles for optimal management after CMSI were also identified, which involved a systematic approach to rehabilitation and the RTP decision. Although CMSI were reportedly difficult to prevent, on- and off-field strategies were implemented by experts to mitigate risk, particularly in susceptible athletes.

**Supplementary Information:**

The online version contains supplementary material available at 10.1186/s40798-021-00364-0.

## Key points

- Experts followed a rigorous process during the clinical examination of calf muscle strain injuries to establish the diagnosis, make an estimate regarding prognosis, and to design an appropriate rehabilitation program.
- Experts recommended optimal management of athletes with calf muscle strain injuries to involve six phases, each with guiding principles and load progressions.
- Injury-specific criteria were utilized in practice to guide the return to play decision and monitor athlete status following the resumption of competitive sport.
- While preventing calf muscle strain injuries was believed to be complex, a hierarchical approach to exercise selection and load management may be useful to inform prevention strategies.

## Introduction

Calf muscle strain injuries (CMSI) are prevalent in elite sports [1, 2] and contribute to the negative impact that any injury can have on team success [3–5]. The burden of CMSI can also be significant, with > 3 months time-loss reported for some cases in American football [6], football (soccer) [1] and Australian Football [7]. Further compounding the impact of CMSI is that athletes are more susceptible to recurrent CMSI and other subsequent lower limb injuries, such as hamstring strains [8–10].

Despite CMSI being problematic in many sports, there is a dearth of research to guide clinicians regarding best practice for the assessment, management and prevention of these injuries [11]. Research into epidemiology and risk factors of CMSI has been the major focus for decades [1, 2, 12, 13]. While these areas form the foundation of prevention models, they represent only some of the areas to consider in injury causation and management [14–17]. In the absence of research about the diagnosis and management of CMSI, sports medicine practitioners have relied upon information provided in commentaries and book chapters to guide their clinical decision-making [18–20], but such resources represent a low level of evidence [21].

Qualitative research is a powerful tool to inform practice and future research when undertaken with a rigorous approach to minimise bias [22]. Qualitative analyses involving in-depth interviews permit complex areas to be explored and evaluated [23]. In sports medicine, integrating perspectives and experiences on injury causation, clinical reasoning/decision-making and injury prevention can guide practice [24] and augment the interpretation of existing quantitative data [25]. Qualitative methods have been used to better understand and develop injury prevention strategies [26–29], as well as identifying current injury management [30]. For CMSI, a qualitative investigation may be especially critical to explore the network of factors that are potentially associated with injury occurrence and to identify possible inter-relationships [17], which may be difficult or impractical to do so quantitatively [24]. Further investigation is warranted given that failed management (i.e. recurrent CMSI) results in a two-week longer average time to RTP [7] and the risk of subsequent CMSI is elevated for months [8, 31]. The aim of this study was to evaluate current practices and perspectives of a select group of international experts regarding the assessment, management and prevention of CMSI.

## Method

### Participants

Participants were required to be expert clinicians working in elite sport and/or clinician-researchers specialising in a relevant field. Potential participants were identified purposefully using publicly available information, the networks of the investigators and identified experts, as well as a review of key research in the field [26, 32]. Using a consensus approach among investigators, potential participants were sourced from different countries, sports and areas of specialisation to ensure diversity in the sample and to minimise the risk of bias [33]. As a minimum they had: (1) postgraduate qualifications in ≥ 1 relevant discipline, and (2) > 5 years of clinical experience in elite sport and/or consulting elite athletes. Recruitment continued until data saturation was reached, which was determined by consensus [23, 34].

### Interview Design

In-depth interviews [35] were chosen to explore the practices and perspectives of experts in the assessment, management and prevention of CMSI. An in-depth semi-structured design enables deeper exploration of participant responses, recognising trends and themes as they emerge [23]. The content of the interview schedule was developed from previous research [30]. A consensus approach was used to refine the interview schedule to be specific to CMSI, including gaps identified in current evidence (BG, TP, ASe, JM) (Additional file 1). The interview schedule was then piloted prior to this study, which enabled further revisions to be made (BG, TP) [22, 36]. It was sent to participants beforehand for approval and was used as a guide during interviews to ensure data were collected on all topics from each participant given that a semi-structured design can result in responses that do not follow a linear narrative [22, 35].

### Procedure and Data Collection

Potential participants were contacted to explain the study aim broadly and were invited to participate. An interview was arranged with those accepting the invitation. Interviews were conducted by one interviewer (BG) with no one else present and were recorded to permit data transcription verbatim[35]. Participants were offered the opportunity to review their responses. Hand-written notes were also used during interviews to assist identifying trends, themes and important points for thematic coding. Throughout, the interviewer’s own experiences, preferences and beliefs were not highlighted as the focus. This study received ethics approval (La Trobe University Human Research Ethics Committee: HEC – 18060). Participants provided verbal consent.

### Data Analysis

Two coders first undertook independent data familiarisation by reading all transcripts in full (BG, TP) [37]. Utilising an inductive method and principles of grounded theory, a constant comparative analysis was undertaken to develop data categories and codes [38] (NVIVO, v.12.1.0, QSR International). Thematic analyses were mixed given the diversity in topics, identifying both semantic and latent themes from data [37]. Initial coding was focused on broad category identification and pattern recognition. Subsequent stages of coding (intermediate, advanced) were used to refine key themes and trends, as well as inter-relationships between concepts and inconsistencies in participant responses [37, 38]. Throughout, memo generation [38] and review [37] among coders was ongoing to ensure adherence to an emergent design [36] and to gauge data saturation [23, 34]. Authors involved were Australian-registered physiotherapists. One (BG, male) completed a PhD investigating CMSI in sport, works clinically in elite Australian Football and has a Masters degree in Exercise Science (Strength and Conditioning). The other (TP, female) is a clinician-researcher with > 20 years of clinical and academic experience, including previous qualitative research, and has also consulted regularly for elite athletes from Australian Football, ballet, soccer and Olympic sports.

## Results

### Participant Demographics

Twenty-six potential participants were identified and invited. Of these, three were not contactable and three opted out. Twenty participants were interviewed face-to-face (n = 9) or using a meeting platform (n = 11). All participants were primary contact practitioners and were engaged in clinical practice across nine countries: Australia, the USA, the UK, Ireland, Norway, Sweden, Spain, New Zealand and India. Participants were primarily involved in managing adult elite (i.e. professional) athletes. Sports of practice were: football (soccer), Australian Football, track and field, Olympic sports, rugby, ballet cricket, and collegiate sports. Half (50%) worked in their clinical roles in elite sport full time. The other half had mixed roles between elite sport and consulting from a university and/or a private clinic. Of the 65% who had research experience, most (69.2%) had completed a PhD. The participants collectively offered a range of expertise, including injury prevention, rehabilitation, clinical reasoning/RTP decision-making, radiology and biomechanics.

### Overview of Results

Interviews ranged between 35- and 98-min duration. Interview data were related to three primary categories that were used to provide a structure for presenting the results: (1) evaluating injury characteristics (clinical examination, differential diagnosis, radiology, estimating prognosis); (2) rehabilitation and RTP decision-making; and (3) injury prevention (aetiology and risk factors, screening and athlete monitoring, prevention programs).

### Evaluating Injury Characteristics

#### Is It Soleus or Gastrocs?

During the initial examination of CMSI, experts valued first identifying the primary muscle involved (e.g. soleus vs gastrocnemius), injury severity and triaging actions (e.g. immediate immobilisation, imaging). To do so, a systematic approach for meeting these outcomes was described (Additional file 2, Tables 1 and 2). Upon examination, gastrocnemius and soleus injuries often exhibited contrasting mechanisms of injury, symptom locations and impairments (Table 1). Although experts reported a greater clinical challenge when diagnosing soleus injuries because symptoms and impairments are at times absent or non-specific until subacute examination: *“It’s just: ‘I pulled up a bit tight,’ ‘I thought I was a bit tight,’ ‘hang on I’m still a bit tight,’ and when we get going: ‘actually, this feels a bit sore now when I try to accelerate,’ or something like that,”* (Expert 16). Experts shaped the initial subjective examination to provide direction about the muscle(s) involved, potential predisposing factors and prognosis (Table 1). Findings associated with gastrocnemius injuries were reportedly well-defined, whereas poorly localised “*tightness*” or “*cramping*” that impedes function and does not resolve was pathognomonic for soleus injuries: *“The ‘shotgun’ bang, you got ‘shot’ in the back of the leg tends to be, well it could be soleus or it could be ‘gastroc’, but more likely ‘gastroc’. And then the slowly creeping, gripping, ‘heart attack’ pain in your calf I think tends to be more soleus,”* (Expert 12).

**Table 1:** Key domains and subcomponents of the initial subjective and objective examination of calf muscle strain injuries

Domain and sub-components	Primary clinical questions and considerations	Key concepts and/or outcomes guiding clinical decision-making
**Subjective**		
*The presenting injury*		
History of onset	*Do you remember how or when it happened?*	Symptoms from soleus injuries are at times cumulative or may not be reported until subacute examination, whereas gastrocnemius injuries are almost always apparent immediately. Gastrocnemius injuries were most common during acceleration, jumping, and sprinting activities whereas soleus injuries were most common during steady-state running or were gradual onset presentations
Self-reported symptoms	*What are your symptoms?*	Frank pain may be more common in CMSI involving gastrocnemius or severe soleus injuries
	*Where are your symptoms coming from?*	Location is often obvious for superficial CMSI. Poor localisation is common for deep soleus injuries
	*How badly are you impacted?*	Self-perceived impairment assists early estimation of prognosis, and triaging actions
*The injured athlete*		
Intrinsic factors	*Have you injured your calf before?*	Details of previous CMSI aided diagnosis and impacted prognosis (e.g. more time may be afforded athletes with a significant history)
	*Do you have a history of any other lower limb injuries?*	Other previous injuries may impact susceptibility to subsequent CMSI pending the impact they have had on exposure or the presence of persistent impairments that affect calf loads
Potential predisposing factors	*Do you currently have pain or symptoms anywhere else?*	Sub-clinical states elsewhere direct objective examination of contributing factors, especially when relevant to the mechanism of injury
	*Have you recently been offloaded from running for any reason or has your exposure to match play or training changed recently?*	Recent interruptions and/or suddenly increased running workloads were a common culprit in CMSI, particularly soleus injuries
	*Have there been any other recent changes to your off-field program?*	Unaccustomed heavy strength or explosive loading may reduce resilience to CMSI involving either soleus or gastrocnemius
*The injury context*		
Extrinsic factors	*What is your sport and how would you describe your playing position/ style?*	The activities performed and mechanical conditions encountered provide direction for diagnosis and prognosis
	*What is the stage of the season?*	Pre-season and early competition periods were associated with over-load related CMSI (e.g. re-exposure to running workloads and intensity)
Other contextual factors	*Has anything changed about the environment you complete training and/or matches, the surface, your footwear?*	Changes in surface and footwear were found to impact the work conditions of the calf. Altered work conditions as a result of these seemingly small adjustments can have a large impact on injury risk due to the high work demands on the calf in dynamic activities and large workloads of elite athletes
**Objective**		
*Observation*		
The calf	*Is a defect observable or signs of a different pathology?*	Defects and/or de-tensioning are evident in some severe CMSI, often involving gastrocnemius
	*Is there a difference in relative bulk?*	Structural differences can correlate with impaired calf function and may predispose to CMSI
Other body regions	*Does the injured side show signs of an unloading pattern elsewhere (e.g. atrophy)?*	Visible difference in synergists might indicate increased calf work demands due to habitual under-loading. This may highlight other body regions to examine for predisposing factors
*Palpation*		
Tenderness	*Which muscle is tender?*	Palpation can usually differentiate muscle involvement and is most obvious for severe CMSI
	*What is the maximum length of tenderness?*	Palpation tenderness over a more extensive length can indicate greater severity, especially for gastrocnemius injuries
Tactile qualities	*Can a defect or focal spasm be located? If so, where?*	Palpable tissue changes can help to confirm the location. Focal spasm often became apparent in subacute presentations of soleus injuries
*Stretch tolerance*		
Non weight bearing	*Is passive dorsiflexion affected?*	Greater range of motion deficits are evident in severe CMSI. Mild CMSI may not show reduced stretch tolerance in Non weight bearing conditions
	*Does altering the knee position highlight the symptom source?*	Soleus and gastrocnemius injuries may be more sensitive when stretched while the knee is flexed and extended, respectively
Weight bearing	*How do the available range and symptom severity compare between knee extension vs knee flexion positions?*	Knee position can differentiate muscle involvement to some extent (e.g. soleus vs gastrocnemius), but severe CMSI have similar weight bearing restrictions regardless of position
*Isolated function*		
Non weight bearing	*Does isometric calf loading elicit symptoms?*	Gross weakness and pain during low-load tests indicates greater severity
	*Do symptoms change according to knee position?*	Greater weakness and symptoms associated with knee position may differentiate muscle involvement
Weight bearing	*What is their calf raise strength (e.g. unable vs double leg vs single leg)?* *Is strength impacted by altering position (knee, ankle, foot)?*	The degree of strength loss is associated with severity and may be position-dependent according to the muscle (knee flexed: soleus; knee extended: gastrocnemius)
	*Are there any other findings if the loading rate is increased?*	Increasing the loading rate could identify subtle differences and bridge the gap between strength and plyometric testing
*Dynamic capacity*		
Plyometric function	*What is the relative capacity for plyometric function (e.g. unable vs double leg vs single leg)?*	Systematically testing graded plyometric tasks can elucidate the injury severity. Severe CMSI will not be able to perform this part of the examination
	*Do the results change whether it is predominantly upwards or forwards?*	Plyometrics involving forward propulsion are the most demanding; CMSI undetectable until this point are likely to be of mild severity
Locomotive activities	*What is their capacity to walk? Jog? Stride? Sprint? Accelerate?*	Relative capacity and the threshold for symptom-onset helps to identify severity. Some mild CMSI can walk and perform some running activities pain free even at the time of injury onset

*CMSI* calf muscle strain injuries

**Table 2:** Restoring foundation calf and lower limb function, and loaded strengthening after a calf muscle strain injury

Guiding clinical principles and primary actions	Key quotes
**Foundation calf and lower limb function**	
Normalise the walking pattern as the first step to normalise movement	*“Get them walking normally as quickly as possible. Get them with normal stretch left versus right as quickly as possible, both gastroc and soleus. And, like I say, ‘load it.’ And it will be more ‘capacity-loading’. Load it safely, pain free, a number of times throughout the day*.” Expert 5
Find the optimal starting point to commence therapeutic calf loading, being specific to the muscle injured from the outset	*“I’m almost exclusively going with load-based or muscle activation exercises. And really the decision is “how much load?” And where possible you’re trying to be as specific as you can with that load. So if it is a soleus-based pathology, then trying to find a way to make sure that they are loading over that area that is injured, they’re not just taking over with other parts of the muscle, or taking over with… you know, if it’s a medial gastroc injury, they’re not just taking over with soleus and you see that the medial gastroc is still just quite flaccid when they’re doing whatever activation exercise it is that you’ve given them.”* Expert 18
	*“Start strengthening probably day 2, day 3. And that will be if you can do band exercises, we’ll do band exercises. If you can do two-leg weight bearing, we’ll do two-leg weight bearing. If you can do one-leg weight bearing we’ll do one-leg weight bearing. And so we try and find where your barrier is every day, and work just below that barrier.”* Expert 12
Prescribe multiple loading bouts per day to offset the likelihood of post-injury sequelae	*“Players come in at 9 o’clock in the morning, and sometimes they are gone by 2 o’clock. Which is ok in some injuries but I am of the thinking with the calf you need to be giving them homework, or you are keeping them with you. If I can load them on four different occasions that day, pain free, safely, then I think you are getting capacity very, very early,”* Expert 5
Use activation exercises to ensure inhibition does not negatively impact higher-load activities	*“Gastroc particularly, just gets very quickly inhibited. So we’ll just do some non weight bearing initially just to get some activation in it, which can be quite hard…. otherwise it could only be your flexors and your soleus doing all of the plantar flexion. So we teach them how to activate just by doing a non weight bearing one…that’s sort of more for the severe ones, but even for the milder ones, just to make sure.”* Expert 19
Foundation exercises can be progressed to include more dynamic actions of muscle–tendon unit	*“Once they can do something like 2 sets of 15 slow and controlled up, on a single leg, then add 1 set of oscillations. We do our oscillations ‘up-top’, so in this position (end range for a calf raise), and then down to plantar grade, and then off a step in dorsiflexion, but that’s done not off reps, it’s done more on time. So 15 s, 15 s, 15 s… when they can do that they go to the more violent ‘drop and catch.’ ‘Drop and catches’ at plantar grade, and then ‘drop and catch’ down in dorsiflexion, for the reason that they spend a lot of time changing into this position for push off. So you have to get them into that range.”* Expert 14
Condition uninjured body regions at the highest intensity possible, while respecting pathology	*“You do not want to let detraining occur in any uninjured muscles. So if you don't have any reasons to stop their other gym exercises, then don't. Keep their same routine. You do not want their general conditioning to lapse as well,”* Expert 9
Use this opportunity to establish the complete injury situation—address potential predisposing and risk factors	*“It will also give us an opportunity to look at any other deficits that they might have. So for example, if there was a quads deficit or there was a posterior glute deficit on that side that was causing them to compromise their triple-extension, then we would look at that. And then we would progressively overload the calf. Looking at getting some endurance back. Looking at the manner in which we do that. Looking at the rest of the intrinsic foot strength. So tib post, peroneals, tib ant. Again, easy to get early value day 1 or day 2 post injury around that while you are respecting the injury itself. And then progressing that on, as appropriate.”* Expert 7
Avoid excessive eccentrics and prolonged passive stretching	*“Range of motion probably takes care of itself. I think that, yeah they might have a painful lunge stretch to begin with but I see that as more an assessment tool rather than an impairment that I need to work on specifically. So I don’t really prescribe stretching exercises as a treatment.”* Expert 19
**Loaded strengthening**	
Have a foundation of single leg calf raise capacity prior to loaded strengthening	*“get some good endurance work—single leg, body weight, and be getting good at that. As a baseline marker it's 20 to 25. Yes, as an end stage or global benchmark I’d like that to be higher. Once I feel they are hitting that in rehab then I will transition them to adding load and less reps. Once we do get through the first cycle of loading, we load heavy. You know, your shorter reps 3 or 4 sets of 6 to 8, and then have a mix of some sets in there where it is probably longer, slower isometric holds.”* Expert 11
Maximising capacity is the first priority	*“I’d potentially start loading them with a straight raise, as in with additional load, and then as they can tolerate their bent knee calf work they can do it seated using a Smith machine. I’d start loading from there. Initially higher reps: 10–15, 10–12 in the first few sessions, and gradually bring that down to your 8’s. And then with increased loading to sort of 3–4 sets of 6.”* Expert 11
As rehabilitation progresses, sports-related strength qualities trump maximum strength	*“While I get their strength up, I think there is that whole strength-endurance component that they’ve really got to build into their rehab.”* Expert 13
Horizontal strengthening is an important piece of the puzzle	*“We also load them in a position in which it mimics most, kind of that ‘leaning tower’ position of running. So rather than just that vertical loading, we are getting them into that ‘leaning tower’ position. So we can get that bar, the bar you might use for a high-bar back squat, and we get them leaning into the racks to do that calf loading.”* Expert 7
Progress loaded strength exercises to restore the range of attributes of the sport—consider work duration, axes, and velocity	“*Work the endurance and work towards velocity over time, as well as building the good old fashioned strength and strength-endurance*,” Expert 6
Shape single leg strength to be the foundation for dynamic exercises	*“We probably wouldn’t spend too much time loading up double leg stuff. We’d go to obviously the next progression up is single leg, is to weight up single leg stuff. And so again, once you can do weighted single leg stuff, then you’d do jumping. And then from jumping to hopping. And then from hopping to running.”* Expert 12
Ensure soleus load tolerance prior to progressing severe or *‘problem calves*	*“where there have been recurrent injuries or where there’s been a higher grade of injury, then we tend to tap more into, particularly soleus strength, as a thread of their rehab. We’ve debated over the journey as to whether that’s a seated or a standing version, and still flip between the two, but we certainly like to have a thread of the strength as well as the strength-endurance that then ultimately underpins that elastic cycle.”* Expert 16
Load compound exercises to complement calf rehabilitation, initially taking care to not overload CMSI dynamically	*“Before they are getting back into training, as a rehab tool get help from glutes, hammies, quads, hip flexors; training all of those things concurrently in rehab. And you can do that pretty early in rehab. Even from day two, just get them to squat, lunges, pulling motions…”* Expert 1

**Fig. 1:**
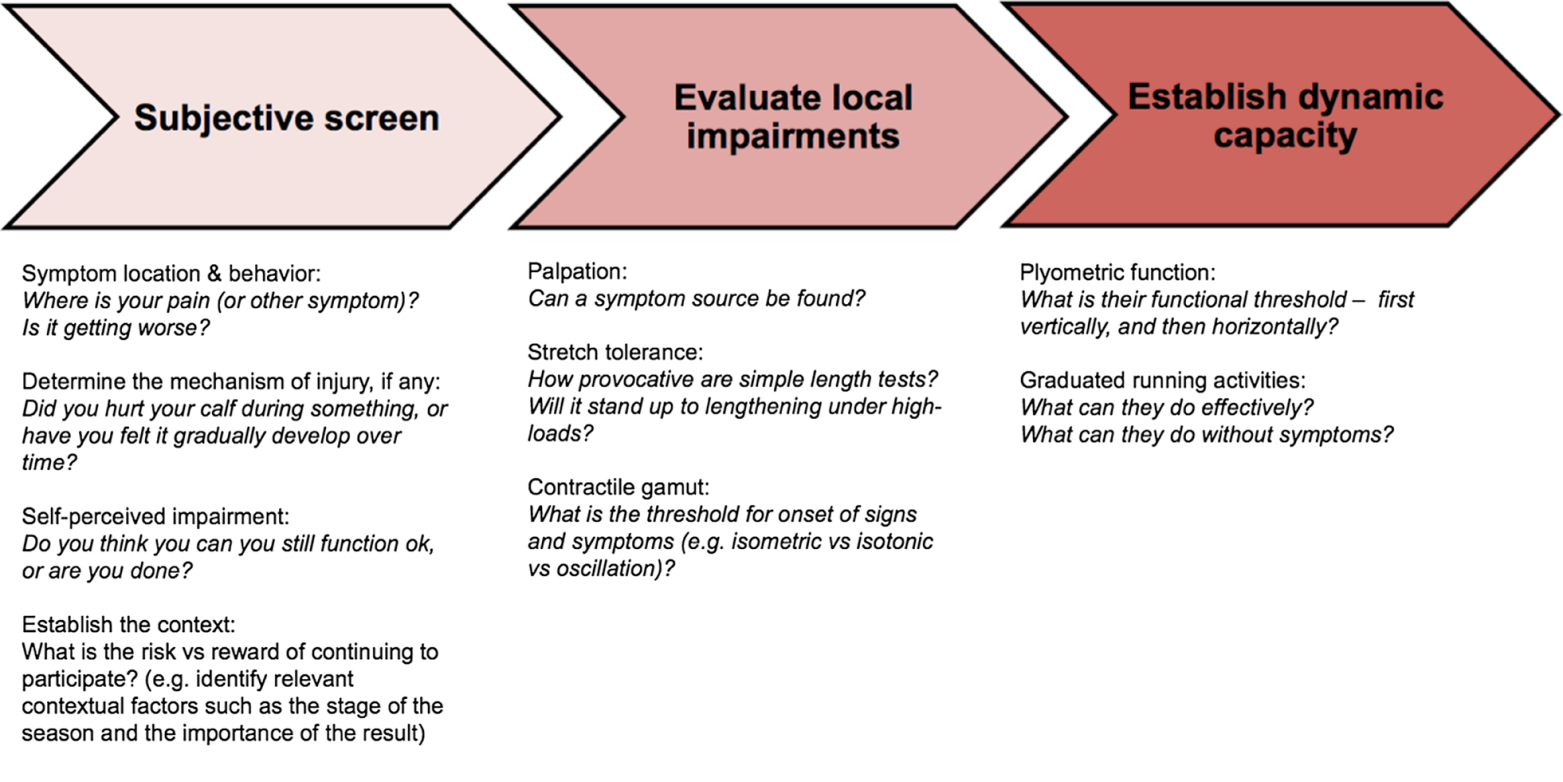
A framework to guide the match day assessment of calf muscle strain injuries based on information provided by experts

Knowledge of the sports-specific epidemiology of CMSI aided examination by helping to identify the likely injury mechanisms and mechanical conditions encountered, which was used to inform the suspected muscle injured. Consistent with this concept, experts reported a higher prevalence of gastrocnemius injuries in rugby, ballet, basketball and sprinters, whereas soleus injuries were reportedly more prevalent in long distance running, Australian Football, and football (soccer). During the initial objective examination (Table 1), experts refined the clinical impression of injury location and severity and directed immediate management (e.g. imaging, continue/cease participation). The calf muscles were observed for deficits in bulk or visible evidence of CMSI—superficial defects were commonly a sign of CMSI involving medial gastrocnemius ruptures at the distal muscle–tendon junction and/or free aponeurosis. Gastrocnemius heads and soleus were palpated to investigate location and length of tenderness. While it was generally accepted that adjusting the knee position during objective testing could help differentiate soleus (knee flexed) vs gastrocnemius (knee extended) involvement (Table 1), experts also highlighted this diagnostic relationship was not absolute. Another important message was that testing for pain provocation was not always reliable for soleus injuries because symptoms such as “*tightness*”, “*cramping*”, or “*awareness*” could be reported instead (Table 1). Match day examination of CMSI was also described to have unique constraints due to time pressure and the *“risk versus reward”* (Expert 10) (Fig. 1): *“On game day, where the bottom line is: is the player done, or is the player continuing to play?”*(Expert 14). Experts agreed that in these situations, while the immediate objectives were to establish the primary pathology and suitability to continue, detecting pathology did not always preclude further participation.

#### If It Is Not a Calf Strain, What Is It?

Experts reported a group of other clinical presentations that could have some signs and symptoms similar to CMSI: direct injuries (e.g. contusion), delayed onset muscle soreness, other lower leg muscle strains and an Achilles tendon tear or tendinopathy. Bony, neural or medical causes were perceived to be uncommon. Here, an understanding of epidemiology was again highlighted as being helpful for framing preliminary clinical reasoning. For example, *‘calf’* symptoms likely arose from different structures when comparing an adolescent to an adult (i.e. CMSI were more common in older athletes). The mechanism of injury and symptom onset (e.g. sudden vs cumulative over days or weeks), and if this was associated with a recent exposure of altered loading, were helpful to inform the possibility of delayed onset muscle soreness or overload pathologies involving structures such as bone or tendon. While common for some CMSI, experts also associated “*shotgun*” presentations with acute Achilles and plantaris injuries. Experts found Achilles ruptures to be easily differentiated from CMSI based on the clinical signs, whereas plantaris injuries were not usually associated with significant impairments despite momentary symptoms: *“The other one we’ve had experience with is if you’ve ruptured your plantaris and you get the big ‘ping’. And if that's ruptured then we just push on,”* (Expert 11). Suspected strain injuries involving other lower leg muscles (tibialis posterior, flexor hallucis longus, flexor digitorum longus, peroneals) could be investigated using manual strength and modified calf raise tests: *“There’s FHL tears that can happen around their origin on the fibula…And tib post, but they are pretty rare…And FDL on occasion, but they’re pretty rare I think too. But I think that FHL is the one that is missed a bit,”* (Expert 13). A lumbar spine examination and neural tension tests were consistently formative components of the differential diagnosis as well: *“There’s that strong connection with the lumbar spine. Often I think they’ve had a niggly back. There is kind of that neural component,”* (Expert 19).

#### Do We Need a Deeper Look? The Role of Imaging ‘Calves’

The decision-threshold for imaging was low in elite sport, but experts still preferred to wait 1–2 days to confirm it was warranted and to gain a complete impression of severity prior to introducing biases inherent in obtaining imaging results: *“We would probably still get an MRI in 80% of cases. But we are certainly not rushing off in that first 24–48 h because you’ll have those guys that do have that ‘DOMS’* (delayed onset muscle soreness) *presentation that you could go and get an MRI and then suddenly be jumping at shadows,”* (Expert 16). In contrast, some experts considered imaging to be contraindicated unless it provided immediate direction for management: *“If it looks like a duck, walks like a duck, talks like a duck: it’s probably a duck. So sometimes it depends a little bit on the athlete and how much catastrophisation will come out of using an MRI,”* (Expert 12). While MRI was used as the gold standard, there was no consensus on a recommended imaging classification to best estimate prognosis for CMSI. Ultrasound was also preferred by several experts to grade distal gastrocnemius injuries and when visualising the interfaces between muscles and compartments. Imaging (of any kind) was useful to obtain an early description of the pathology, but over time the rate of functional progression provided the most valuable prognostic information: *“We are always mindful of damage versus function: tissue damage versus function. We don’t mind scanning, and saying, for example: ‘you have got a low grade calf strain to the soleus. We have seen players not miss, or miss 2 to 3 weeks, so at the moment anything is possible,’ and that becomes the prognosis,”* (Expert 15). Variation in prognosis also occurred in radiologically severe CMSI: *“Whilst I would have cases that substantiate disruption to aponeuroses that are perceived to be important for load-bearing, and that being an indicator of poor prognosis, I’m sure we probably have cases where we’ve also shown damage to those tissues that have gone back ok because we’ve just pushed them based on their clinical signs anyway,”* (Expert 18).

#### “Ok, It’s a ‘Calf’… When Can they play again?”

**Fig. 2:**
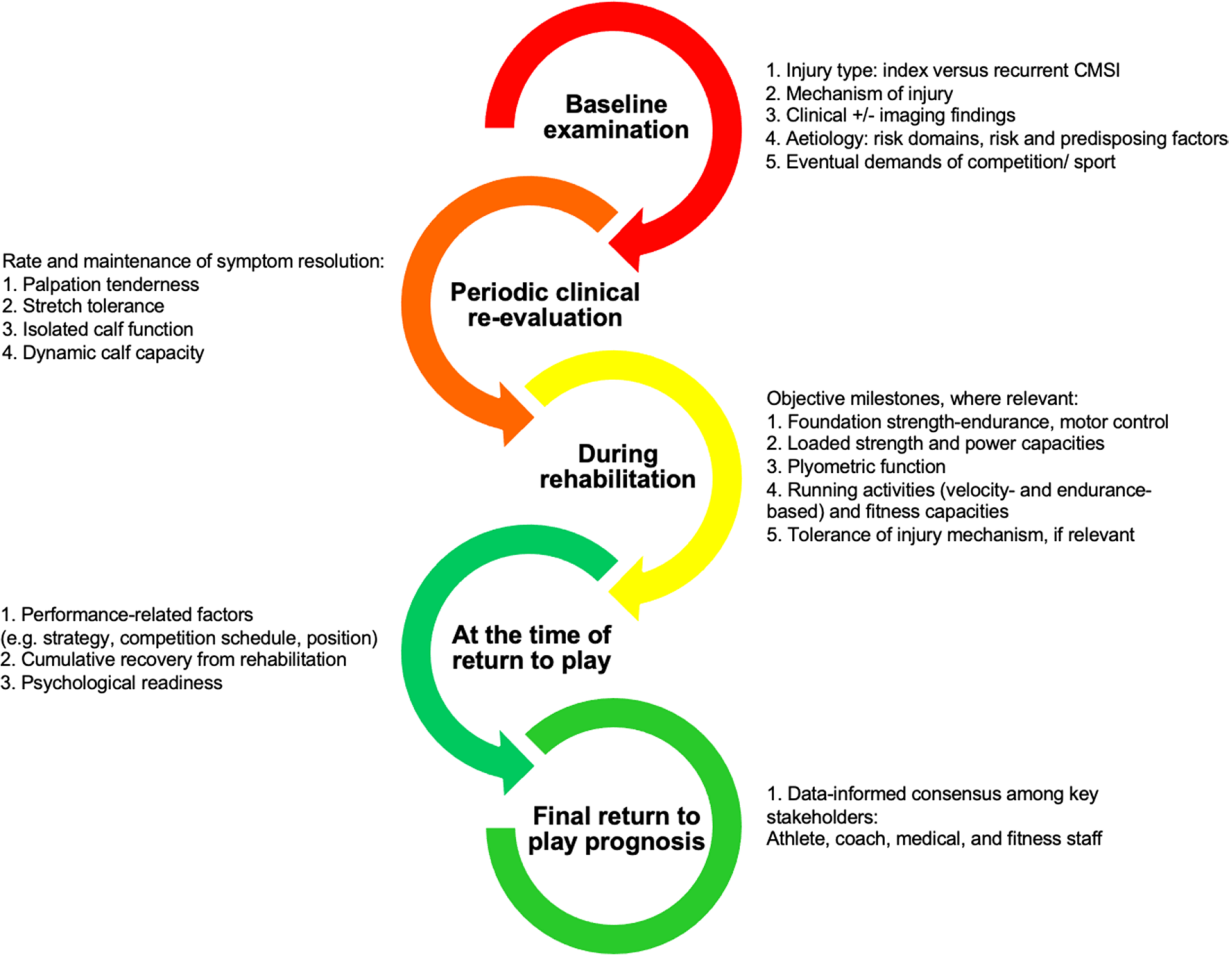
Evaluating prognosis after a calf muscle strain injury. Numbered dot points refer to the primary themes and/or concepts that influence decision-making at each stage

A staged approach for accurately determining prognosis after CMSI was identified from information provided by experts (Fig. 2). Experts perceived the value of staging the approach to be three-fold: (1) recurrence due to overly aggressive rehabilitation or premature RTP clearance was less likely, (2) unnecessarily conservative RTP time frames were avoided because data-gathering is ongoing, and (3) performance-related factors are able to be considered and planned for.

*First, the crystal ball: Estimating recovery at baseline:* Experts based baseline prognoses on injury severity. This was best graded by combining information about the injury circumstances, function and imaging (Fig. 2). While experts recognised this was a necessary part of disseminating information among stakeholders, early expectations were best stated broadly (i.e. none vs short vs extended) to allow for refinement based on clinical progress. Periodically screening athletes for predisposing factors for subsequent injuries (recurrent CMSI or other) was another important theme, since this information influenced prognoses irrespective of pathology: *“I have seen players that have ‘one week lesions’ on a scan miss six. And I have had other players who have, you know, what looks like a three or four-week injury on imaging play…I think it comes back to those internal factors, the ones that can play with them are strong, good athletically, minimal soft tissue injuries, young. Whereas if you get a smaller lesion in an older player, with no strength, poor training age in the gym—they can’t cope if they don’t have the architecture to support that lesion. And taking a holistic approach, and really knowing them inside out. Knowing their training background and their injury history,”* (Expert 1)*.*

*Second, the magnifying glass: Refining the prognosis during rehabilitation*: Once rehabilitation commenced experts utilised functional progression milestones and athlete monitoring data to refine the prognosis (Fig. 2): *“What trumps MRI is the clinical progression: the recovery of strength, the recovery of range of motion, the ability to progress through clinical milestones, decreased swelling, decreased pain, muscle activation, resolving strength. Those to me are a lot more important,”* (Expert 10). Early focuses for experts were the rate of resolution of pain free walking (*“players saying ‘I felt a big rip, a big pop,’ and you’ve got someone who can’t walk pain free until after day 10: that's a 6 to 8 week calf before you scan it,”* (Expert 15)), palpation tenderness, stretch tolerance, single leg calf raise strength and plyometric function. Calf capacity during exercises involving high relative load and loading rates, and running milestones, provided the most direction as rehabilitation progressed (Fig. 1). Even more careful attention to meeting objective milestones was afforded cases of multiple recurrences or at identified time-points where risk was perceived to be elevated, such as re-commencing running. Detecting potential risk factors or other clinical findings, including impairments in other body regions, that could increase susceptibility to subsequent injury (recurrent CMSI or other) were also considered by experts when deciding the rate at which rehabilitation progressed.

*Last, the microscope: Confirm the prognosis at the time of return to play—not before:* Experts encouraged the final RTP decision to be made by consensus (Fig. 2). Rather than pathology being the exclusive focus, performance-related factors were cited to be a common justification for changes to prognosis at this late stage, which varied greatly among sports. For example, a lack of competition readiness due to residual fatigue or limited training chronicity. Experts also refrained from routinely re-imaging prior to RTP to *“confirm healing”* as a perceived safeguard against recurrence: *“If we accept that clinical findings are actually better for us prognostically than MRI, sometimes the MRI can overly cloud your judgement, and I think that applies to calf injuries more than it does for hamstrings and quads, and other things,”* (Expert 16).

## Rehabilitation and Return to Play Decision-Making

### Overview of Management

**Fig. 3:**
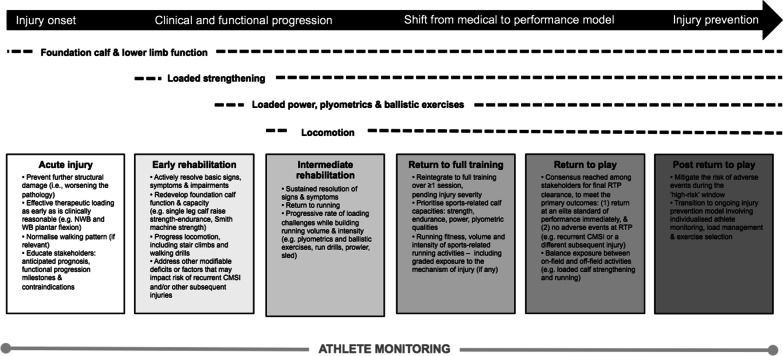
An overview of the optimal management of calf muscle strain injuries described by experts. *NWB* non weight bearing, *WB* weight bearing. *Note* Mild CMSI that do not result in time loss or have a prompt RTP do not require the complete process outlined

Over the course of rehabilitation, the clinical reasoning of experts transitioned from a predominantly medical mindset to prioritising performance, and then preventing injury after RTP. While expert responses highlighted best management is highly context-dependent and strongly influenced by athlete intrinsic characteristics and external factors, exercises and load were progressed in a sequence that reflected six management phases—each of which were embedded with guiding concepts and principles experts found useful (Fig. 3). Similarly, successful management was collectively perceived to be determined by three outcomes: (1) RTP as soon as possible, (2) restoration of athlete performance to the expected level, and (3) no adverse events (e.g. a recurrence or other subsequent injury).

### Rehabilitation Principles

#### Early Loading and Foundation Calf and Lower Limb Function

**Fig. 4:**
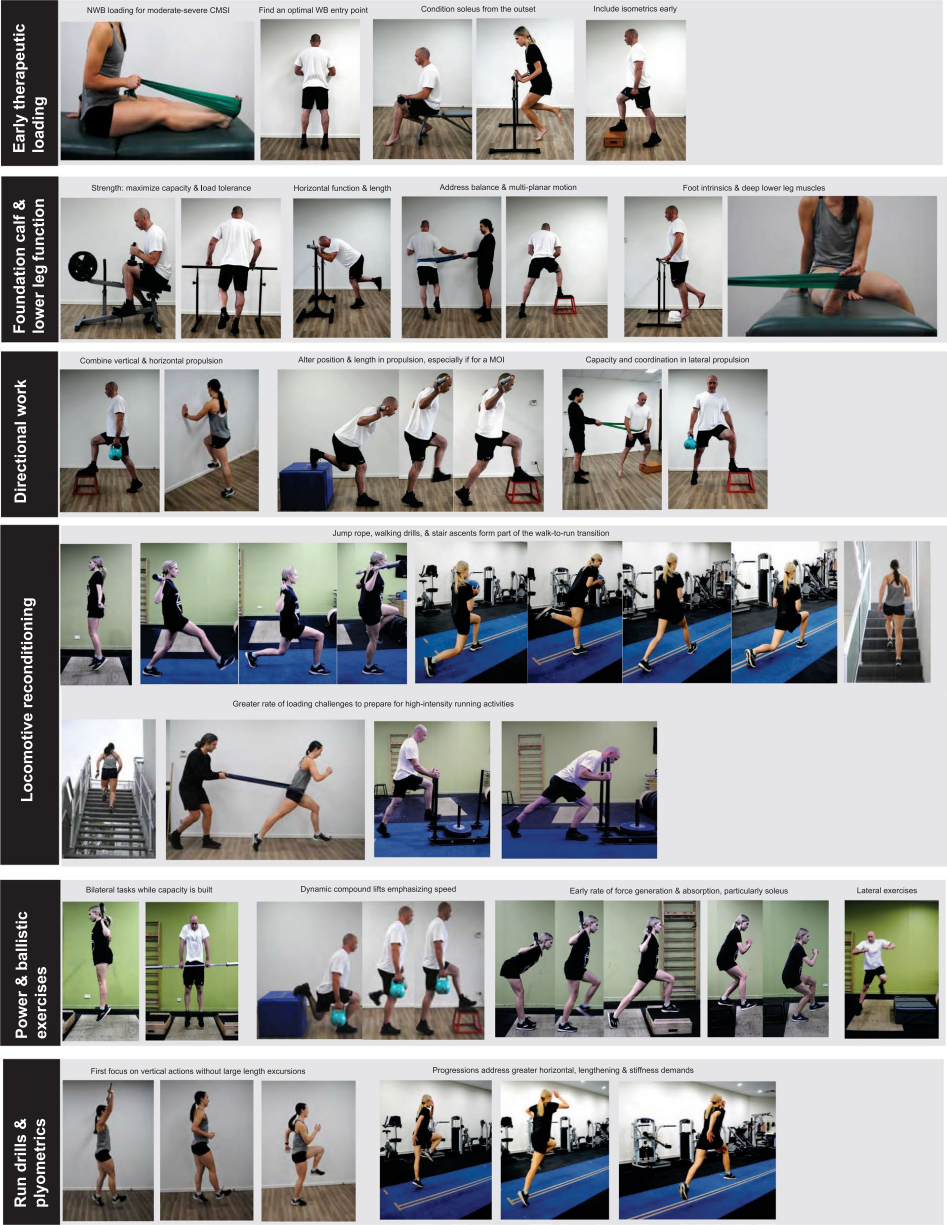
Examples of exercises and principles experts used to guide the rehabilitation of calf muscle strain injuries. *NWB* non weight bearing, *WB* weight bearing, *MOI* mechanism of injury

Experts perceived early loading to be therapeutic by fast-tracking resolution of the basic signs, symptoms and impairments associated with CMSI (Fig. 1, Table 2): *“The most important thing is getting therapeutic loading started as soon as possible,”* (Expert 18). Exercise selection and precise load parameters varied among experts—with prescriptions most influenced by injury severity and the muscle injured (Fig. 4: line 1, Table 2). Isometric (*“you might find that isometric loading at certain angles, or at certain muscle–tendon unit lengths, is less symptomatic in the early phase of rehab. So therefore that’s the loading you do,”* Expert 18) and calf raise variations were common early loading strategies (Fig. 4: line 1). Experts encouraged prescribing single leg calf raise exercises as soon as tolerated to restore a foundation of muscle capacity (Fig. 4: line 1 and 2): *“We’ve got to be single leg heel raising, really, straight away. So we can go from an isometric, which is usually only for a day just to get their confidence. I don’t waste time with bilateral, I think they just cheat, so I would rather them just do an isometric, mid-range, or a comfortable range, and then small range isotonics, then full range as soon as they can, even if they can only do 2. I’d much rather them do that than do 100 bilateral,”* (Expert 19). Prescribing multiple loading bouts per day (Table 2) and progressively loading throughout the full range of motion (or muscle–tendon unit (MTU) length) were also perceived to promote faster functional progression and reduced the risk of post-injury sequelae such as atrophy and inhibition. Directional work (horizontal, lateral) was another important consideration for retraining weight bearing function for experts returning athletes to sports involving acceleration and cutting, such as rugby, or if the injury involved these mechanisms of injury: “*We get them strong in terminal, inner range, plantar flexion. We do that almost like a motor exercise, where again, we get them into the ‘leaning tower’ position, with their good leg resting on a small stool, to get them balanced, we will then get them come up into terminal, inner range plantar flexion, and then get them to lift off the front leg while maintaining that 45 degree lean, or whatever angle it is,*” (Expert 7) (Fig. 4: line 1–3). Experts valued cueing single leg calf raises strictly because these exercises were viewed to underpin advanced function: (1) perform work along the axis of the second metatarsal, (2) maintain neutral foot and ankle positions throughout the prescribed range, and (3) control the loading rate (e.g. 1 s: 1 s). Experts identified three cardinal signs of poor calf muscle recruitment and/or function—the *“sickle sign”* (Expert 14) (i.e. progressive inversion and adduction), *“clawing the toes”* (Expert 7) (i.e. over reliance on the deep flexors), and reduced eccentric control (Additional file 2: Figs. 2 and 3). Kinetic chain function was a particular focus during exercises involving horizontally-directed force (Fig. 4): “*Poor athletes will try and come back into some extension*—*so they will come back into lumbar extension, or they won’t be ‘stiff’ in their glute, and quad, as well as their calf. So again that would be something that we would look at, alongside or before they get to the loaded strengthening phase,”* (Expert 7).

A novel concept mentioned by several experts was early exposure to more dynamic MTU actions during simple calf raise exercises (Table 2), which was perceived to benefit CMSI involving disrupted aponeuroses by encouraging tissue dynamics between contractile and elastic elements in low-load conditions prior to dynamic exercises. Retraining balance and proprioceptive function, the foot intrinsics and deep lower leg muscle exercises (Fig. 4: line 2) were described to have greater utility in *‘problem calves’* (i.e. severe or multiple recurrent CMSI), prolonged time to running, or impairments associated with previous foot and ankle injuries. Proximal function and addressing impairments that could impact subsequent injury risk were prioritised universally (Table 2).

#### Loaded Strengthening

Smith machine and seated calf raise machine (Fig. 4: line 2) exercises were a common starting point for loaded strengthening, which experts integrated after an early benchmark of single leg calf raise capacity was demonstrated (Table 2): *“We use the Smith Machine a lot, with weights or weight vests. We progress from a flat surface to stand on an incline to increase the range of motion with those kinds of exercises too,”* (Expert 10). Experts progressed loaded strengthening parameters to reflect sport demands. For example, strength-endurance in sports that involve prolonged running and work (e.g. football (soccer) and Australian Football) versus maximum force-generating capacity for shorter durations (e.g. rugby, sprinters) (Table 2). Although irrespective of the muscle injured and the sport, soleus load tolerance was perceived to be essential for all CMSI prior to introducing dynamic exercises (Table 2). Another emerging theme from several experts was that a failure to consider horizontal and lateral (Fig. 4: line 3) capacities was a shortcoming of conventional strengthening after CMSI, particularly in sports that involve rapid acceleration and cutting. Experts also used more extensive exercise interventions for ‘*problem calves’*. Heavy isometric strengthening at various MTU lengths, augmented eccentric overload (particularly for rugby players involved in the scrum), and altering whole-body positions (e.g. ankle dorsiflexion, knee flexion, trunk lean) (Fig. 4: line 2 and 3) were strategies cited to maximise relative load tolerance across a range of activities and resolve residual strength impairments, while addressing each contraction mode: *“Just to put on record, “how do you train it if its ‘tendon’ versus not?” We just train all elements anyway. So when people say for a hamstring, “are you going to focus more at the hip or the knee?” Or “Are you going to focus on the soleus or the gastroc?” Or “isometric or through range?” Our approach is to train it completely…we are less directional, and more just confident that you’ve got enough time. We have enough time to just cover it all, instead of just trying to work out: “This one really needs eccentric, this one needs isometric, this one needs…” And so on. We just cover it,”* (Expert 15).

#### Loaded Power, Plyometrics and Ballistic Exercises

After meeting preliminary strength benchmarks, dynamic exercises were included (Table 3) to gradually re-expose the calf MTU to actions utilising the stretch–shortening cycle (SSC). These exercises were perceived to meet two primary objectives: *“The first bit is about volume and the ability to contract and to work. The second is about that rate of force development or spring, because in the end it comes back to that rate of loading rather than the total force,”* (Expert 9). Mixed approaches (i.e. loaded and unloaded) to redevelop the capacity to execute and withstand SSCs were reported (Fig. 4: lines 4–7), which underpinned tolerance of sports-specific field-based activities that involve greater loading rates (Table 3). Experts tended to first prescribe dynamic exercises involving predominantly vertical actions, followed by exercises involving greater horizontal, lengthening, and stiffness demands (Fig. 4: line 7). Two main exercise streams were subsequently identified: (1) repeated SSCs over small length-excursions (or pseudo-isometric), associated with a rhythmic MTU action (e.g. single leg pogos), and (2) single or several SSCs over larger length excursions (e.g. single leg countermovement jump, forward hopping) associated with an accelerative MTU action. A novel concept was the need to develop *both* instantaneous and repeated power of the calf MTU for sports that require both of these qualities, such as Australian Football, soccer and long sprinters (Table 3)—which was described to present a clinical challenge due to the competing adaptations these attributes require. While most CMSI could tolerate elementary plyometrics quite soon (e.g. jump rope), experts identified that ‘*problem calves’* required a more comprehensive work-up culminating in advanced exercises utilising inclines, stairs, and different surfaces: *“Do their plyometrics up on an incline… they can also do their jumping and drills up them too. Like the ‘rudiments’, the broad jumps…”* (Expert 1).

**Table 3:** Loaded power, plyometrics and ballistic exercises, and running rehabilitation after a calf muscle strain injury

Guiding clinical principles and primary actions	Key quotes
**Loaded power, plyometrics and ballistic exercises**	
Once load tolerance has been shown during strengthening, begin rehabilitating dynamic muscle–tendon unit actions	*“Isolated strength progressions will overlap and entwine with slowly getting them moving more dynamically. So getting them started a couple of days after you start loading them with decent single leg strength work. Once they start adding good loads then they can definitely start to add in more advanced dynamic work.”* Expert 11
Use mixed approaches (loaded and unloaded) to restore elastic function prior to running	“*You think: ‘Gee this guy looks good,’ and then they go out and start running, and we have even manipulated their running depending on what tissue they have injured, but then they break down. I think it’s perhaps because we have restored strength, length, endurance, but we haven’t considered ground contact times in rehab with plyometrics and explosive exercises*,” Expert 1
‘ *Power-endurance’* is often an important attribute at RTP: build both instantaneous and repeated power capacities	*“I think you really need to respect running for what it is: how much repeated force goes through your body, the elastic properties you need to run. And I think people might take that for granted—these abilities you need to develop in rehab before you can tolerate running demands.”* Expert 1
Dynamic loading can bridge calf capacities developed during heavy strengthening and field-based activities, as well as re-exposure to the mechanism of injury (if any)	*“If you can’t hop well then you can’t sprint. It is just that simple. You don’t have the tissue capacity. You don’t have the elastic properties…it just means that their risk of injury is probably higher, in my experience, of re-injuring that calf.”* Expert 2 *“The issue with calves is that they’re the first point of loading in our kinetic chain, and this is the thing that’s often lost in rehab, you know. So whereas you can accommodate stuff more proximally, there is nowhere to hide with the calf… that is why the problematic ones are really problematic.”* Expert 13
**Running rehabilitation**	
Begin the path to running reconditioning early using low-load walking drills and exercises	*“As soon as they are tolerating isolated calf work, I will start to add in some more dynamic work. So I might do things like a toe walk or a ballet walk up on their toes, and you can add in a couple of kettle bells or dumbbells into their hands. And then start progressing into some more dynamic movements. So before I start adding in true foot stiffness, advanced plyometric type drills, I will often get them to do some ladder-based drills, maybe ‘under-overs’, so there is less vertical explosive force. Something like an ‘ecky-shuffle’; side skips, grapevine on their toes…”* Expert 3
Gradually rehabilitate locomotive capacity—CMSI are unforgiving	*“There’s no warning often with a calf in terms of a recurrence, and so there is a bit of risk-aversion to how to deal with calves in rehab. Whereas a hamstring, generally they have a little bit of ‘awareness’ or a little bit of tightness and you can pull back from there. Calves in my experience feel ‘good, good, good’ and then ‘no good.’ And so there’s no warning. There’s no ability to modify the session. So I think you almost need more confidence that its ready to go before you start exposing them,”* Expert 16
In the lead up to running emphasise run drilling and technique to smooth the transition and to ensure coordinated use of strength and power	*“focus on, or use imagery, to minimize their time on the ground. And just put them in better positions….I think a lot of people who run ‘out the back’ have a lot of calf-Achilles issues.”* Expert 1 *“Lots of ‘pitter-patter,’ jump-land-react…it’s all the A-skips, the B-skips. I think you are teaching the coordination of the muscle again too because I think, when you see calves ‘go’ they often ‘go’ at low level, or stride pace, barely changing direction, it can’ t always be ‘we have exceeded the tissue capacity of the calf.’ Part of it has to be: ‘if it tries to fire at the wrong moment, when I put 90 kg and the rest through it, it re-injures.’ So there’s a lot of timing drills, and patterning and so on, in early rehab for us.”* Expert 15
Capacity must be *high* prior to running, accept a slight delay in return to running for CMSI compared to other muscle strains	*“The loads in the calves are poorly understood—the very fact that running is essentially hopping from one leg to another, and I think that’s really poorly conceptualized by clinicians. So once they do all the slow training, and then go to run, bugger me they fail! So I think really getting the message out about how much load the calves absorb during normal running, I think that’s a ‘biggie.’ In fact I think it is one of the ‘biggies’ to be honest…how long do we wait before we start mobilizing? I think of the calf and our perfusion below the knee is less than what it is in other tissues. That’s something that the plastics guys talk about, and the orthopaedic surgeons talk about it too. We, you know, depending on the severity, but anything grade 3 then I am generally waiting 7 days or so before starting to load them, 5 to 7 days before doing too much. But mind you, once they can walk, I’d put them in a heel lift, and just start walking. But for some I’d extend that out to 7 to 10 days for a grade 3.”* Expert 13
Monitor functional milestones to determine readiness to run, and then use the first runs to test the waters—taking care to avoid too much “*plodding*”	*“If you can’t do 20 repetitions of single leg hopping without having symptoms there is no way you should go running,” Expert 10; “Usually do 8* × *80 m and get them to sit or stand between reps. I don’t get them to walk back between reps because that is ‘time on the legs’. And then we will try to aim for 2 to 3 blocks of that, with enough rest between sets that they feel like they can run again.”* Expert 1 *“We start at about 60%, so that’s 4 or 5 m per second. The really slow running is effectively all calf work, whereas once you get your pace up a bit, you are starting to get some contribution from the more proximal regions. And again, you can mine from Tim Dorn’s paper* [39] *how long their contact times are as their pace increases, and so I think you try to keep their contact times not too slow.”* Expert 13
Progressing running volume requires the most careful attention for sports involving large running workloads and for *‘problem calves’* involving soleus	*“For the calf it is probably more overall volume and what’s in that volume, and how that plays a role. Whether its high ‘accel-decels’, or high ‘B3’ or moderate speed running. So early on…our running would maybe just tick over on the edge of the 18 km/hr, but not try to give them too much in that 18 to 24 dose in the first one or two sessions, and then introduce that from there. But just always keep an eye on the overall volumes of it.”* Expert 12
	*“Why can soleus be difficult? I guess it has a slightly different role to gastrocs. It is a lot deeper and you can't poke it or feel it as easily, or as obviously, as some particular issues. I think maybe it has a slightly different action. I think it is ‘on’ more often—so it might be prone to fatigue, so endurance might be an issue. Strength-endurance might be and issue with it. Often you can do ok for a short period of time, and also in terms of the speed of running. I know as speeds increase loads exponentially do as well and at higher speeds…so again unless you’re going full gas for a long time you can often compensate and get by. So I think that is a problem because people cannot go full gas in training and in ‘game sims’, and they don’t get anywhere near the same level of fatigue, or the same kind of uncoordinated desperation in running, particularly with defensive running, that, once you get on the field, and you’ve got to do some defensive running late in the game and you’re knackered, you can’ t’ pull up, you can’ t go 90%, you go full gas and then ‘bang’….”* Expert 16
Address the range of running capacities needed to perform in the sport at RTP, and to be resilient to recurrent CMSI	*“Acceleration is probably the big one, and then high-speed running. So, you know, your calf work plateaus at about 7 m per second, so once you can sort of build up to that speed, running faster doesn’t actually make any more work on your calf. But going from standing still to as fast as you can in a few steps—that’s the thing that really loads it up. So we will gradually build up speed, but the thing we are probably most careful on is max accelerations. Now we will start, once you can get through the first session of running, we’ll start accelerations but, you know, it will be from a standing start to a jog over 5 or 10 m, so it’s not explosive accelerations. Slowly, slowly, increase the velocities. Once the velocities are higher we will decrease the distances.”* Expert 12 *“We have some timed agility grids we progress through. Some ‘random’, so uncontrolled, unplanned agility type work, and that’s a conditioning thread that works through the rehab plan as well, because obviously we are not dealing with a linear sport.”* Expert 16
While volumes and intensity are built, running attributes can be fast-tracked using reconditioning methods	*“I see their reconditioning as moving loads through range, so making it a bit more functional, rather than just doing a calf raise up and down. I’d use banded catch ups or Prowler, or Prowler catch-ups, or some sled walks….it is important because you are training your acceleration or your horizontal velocity movements. You are applying greater force through the ground in probably more, for want of a better word, ‘functional’ based positions where you’re bringing in extension or triple extension positions, rather than what we find with seated calf raise and these sorts of things. It is a nice load progression that replicates closer to where they are going with what they do in the game… and it gives me greater confidence if they can push greater load through in those sort of ranges and positions, that they are going to tolerate greater loads and forces that matches up with their match-like movements.”* Expert 11
Devote time to building tolerance of and exposure to the mechanism of injury during field-based rehabilitation	*“Bear in mind how it happened too. If you’ve got a guy that has told you, quite specifically, that they were jogging backwards and they’ve gone to suddenly explode forwards as hard as they can and felt a grab in their calf—if that’s the mechanism of injury, that might be something you’d wait a little bit till toward the back end of the rehab process, and also include it as a key focus point in your functional rehab progressions versus a jump-land guy who, you know, say a ruckman who felt a grab in his calf landing from, or taking off for, a contest or something. And maybe the jump-land activities is a focus point for you when you rehab, and perhaps it is something you introduce towards the back end of the process.”* Expert 18

#### Locomotion

**Fig. 5:**
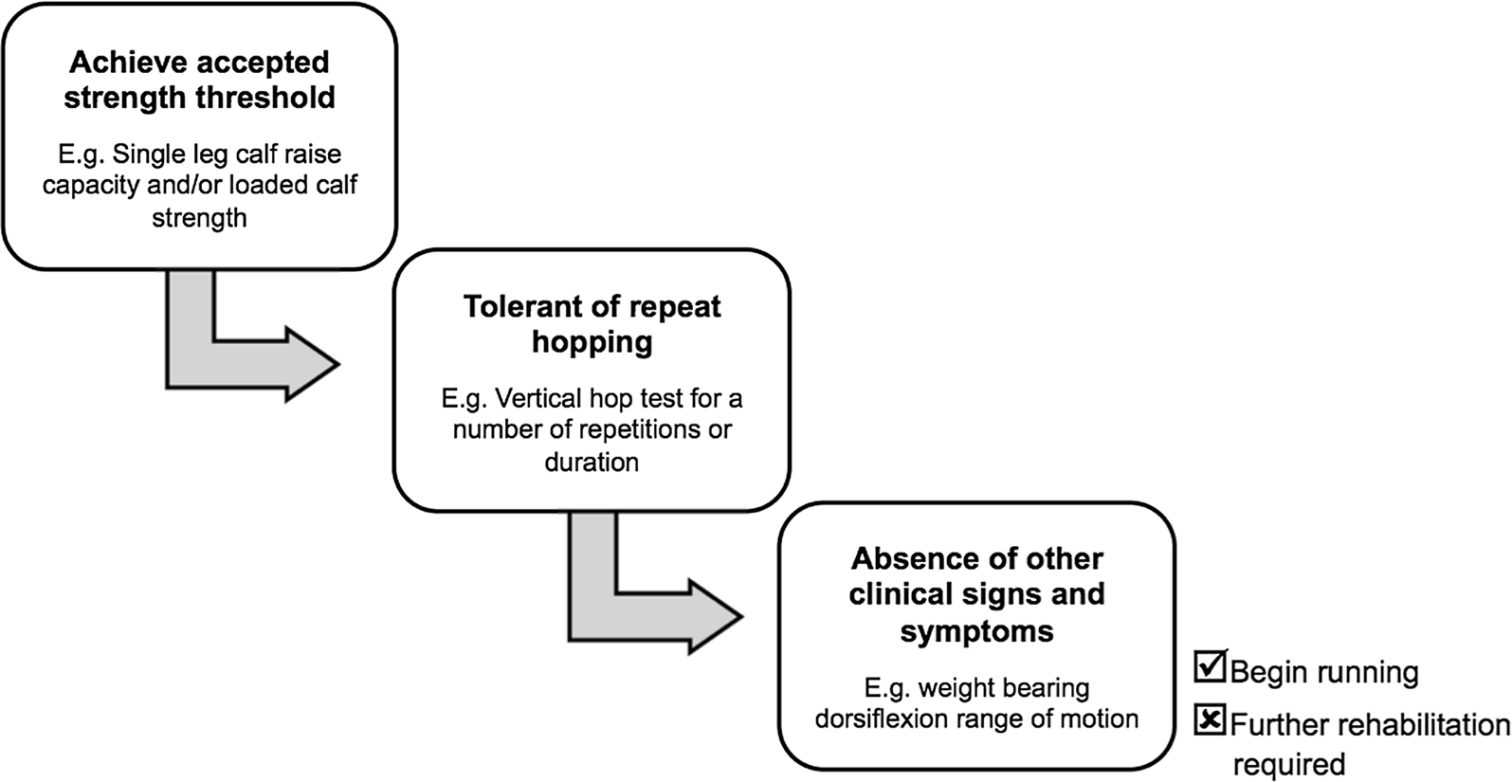
Determining readiness to run after a calf muscle strain injury

Experts perceived testing readiness to run after CMSI to be a key clinical decision due to the large work demands the calf muscles will face during running. Strength, hopping capacity and the absence of other clinical signs and symptoms were the three primary elements of the clinical process identified from information provided by experts (Table 3 and Fig. 5). A* “competency-based”* (Expert 12) approach was endorsed given that running prematurely was cited as the leading cause of early recurrent CMSI in the experiences of experts. A prevailing concept was that a more comprehensive build up prior to running enhanced outcomes because it did not necessarily prolong RTP time frames but mitigated the risk of recurrence (Table 3).

Experts also utilised a novel subset of reconditioning exercises intended to facilitate the return to running by gradually redeveloping locomotive function and capacity (Fig. 4: line 4 and 5). Low-load locomotive reconditioning exercises (Fig. 4: line 4) were prescribed quite soon after CMSI, such as stair ascents (*“We use stair walks in our transition from walking to running. We are lucky enough to have five flights of stairs. We might have them walk up the stairs and then catch the lift down to mask them from the eccentric load,”* (Expert 12)), walking drills and resisted walking (*“A lot of ‘bear crawls’… Pushing on the track as well, doing some lunge-walk drills through that range,…some exercises, you know, where we are aiming for a 45 degree body position like with the ‘wall A-drills’, but also some calf raises at that position to make sure I’ve got some good contractile function as well,”* (Expert 6)). Run drilling and technique exercises were also integrated in the lead up to running (Table 3, Fig. 3: line 7):*“Gradually, I’ll work through some full drills, track drills. So you work through ‘A’ drills, ‘B’ drills, skips, marches, lunge walks, you know those sorts of things. With a bit of resistance, I will do a bit of resisted stuff in the first instance. And then gradually I’ll start them off running, and I will tend to run them, we tend to say a limit of about 5 m per second on their running for the first week,”* (Expert 9). Some experts also felt that movement efficiency and coordination during these exercises were common oversights, but could add value if identified to be a potential contributing factor and for athletes with a low training age (Table 3).

Six ‘*rules of thumb’* were identified from information provided by experts to guide running rehabilitation after CMSI: (1) initially run on alternate days, (2) avoid *“plodding”* early, (3) do not progress volume *and* intensity on consecutive days, (4) schedule off-field exercises (e.g. loaded strengthening) after running, (5) shape running progressions to meet the demands of the sport—don’t overshoot with excessive volume, (6) avoid sudden changes in conditions, such as the surface and footwear. Learning from past mistakes, experts preferred to avoid prolonged, slow continuous running (i.e. “*plodding*”, “*go and jog 5 laps*,” Expert 4) during early running rehabilitation because it had been found to predispose to recurrence for CMSI involving soleus. Greater success was reported when prescribing submaximal run throughs (Table 3). Over time, running rehabilitation involved gradual exposure to greater volume and intensity, with prescriptions aligned to rehabilitate the entire spectrum of activities performed in the sport, including sprinting, cutting, and acceleration, as well as the mechanism of injury. Experts also advocated taking additional care when building volume for athletes that are rehabilitating a soleus injury in order to mitigate the risk of fatigue-related recurrence, especially if they are returning to a sport involving large running workloads (e.g. soccer, Australian Football, distance runners) (Table 3): *“The last thing we tick off is the endurance…it is all very nice ticking off the sprinting and the high-speeds, building their confidence. But at some stage in the match, and in training, they are going to have to cover 12-13 km, and a lot of that is jogging. But it is the last thing we ‘tick off’, as opposed to the hamstrings and the quads, which is the first thing we ‘tick off’,”* (Expert 5). Experts were mindful not to progress running intensity too quickly as well: “*In those first few sessions I am still not going to get them going out sprinting. Because you’re still going to get very high forces and it is very energy storage and release with the higher-level running,”* (Expert 12).

*‘Problem calves’* involving soleus required greater attention to building running capacity (Table 3). To complement this process, many experts progressed reconditioning exercises to prepare the calf MTU for the high relative load and rate of loading demands of the most dynamic activities: “*Sled, fast sled pushes, scooter, stair bounds, and other things before then going into the more functional accelerations, before getting into the rapid change of direction stuff, so then you are preparing them more for their final phase of their sport, which is getting them into training and then ultimately readiness to play,”* (Expert 13). Locomotive reconditioning was described to bridge conventional gym-based exercises and field-based activities. Alternatively, neglecting to restore function at these higher loading rates was perceived to be a culprit in failed management (Table 3): “*We almost go to a different paradigm of loading quantification. The big thing is, it is partly about the tension, but then it’s the rate of force application, and this is something that is not used in anything at the moment. Except for maybe a bit in bone loading. I think it’s something that, as clinicians, we need to expand our paradigm*,” (Expert 13).

### Reaching an Optimal Return to Play Decision

Experts felt the best RTP decisions were reached by consensus among stakeholders, driven by the question: *“What is the acceptable level of risk that the player returns at this time?”* (Expert 10). A clinical checklist to aid determining readiness to RTP after CMSI is shown in Table 4, which is based on information experts found to be useful. Prior to RTP, experts used the return to full training phase to gauge load tolerance and functional improvement (Fig. 2): *“Start to drip them into drills. Generally if it is a big, wide open running drill, full field, we will happily put them in once they’ve gotten through certain things in rehab. If they haven’t demonstrated full acceleration, then some of the shorter, smash-in type things we will keep them out of. When they have done those in controlled environment, we will then start to put them in,”* (Expert 12). During the RTP phase experts were most guided by exposure to sports-specific activities: *“You need to make a decision about: “Well, this guy plays this sort of role, he’s an explosive marking forward, and what does this player do in a game? And how many times do I want to see that at training, those sorts of activities, before I’m happy to know that he’s done that, he hasn’t reacted adversely to it…” That’s the sort of thing that we’d work* *through. But, at a bare minimum, guys have got to get themselves through at least one main training session where they’ve done everything, and they’ve done all their position-specific activities, and have been fine. They haven’t been apprehensive about anything in that session. Their GPS data mimics what you would normally expect to see from that type of session. And yeah, they’ve pulled up fine the next day. But sometimes you might broaden that more and say: “No. I don’t want just one training session. I want to see 2 or 3 because it’s a more extended injury.” Or they’re coming back the second time around after an exacerbation of an initial injury. Or maybe they’re a player that’s just had a lot of trouble from time to time. But for a simple calf injury that’s taken 2 or 3 weeks to settle down, well you’re not going to make them train for 2 or 3 weeks before they play, especially if they’re an important player. Otherwise you’re going to be out of a job pretty soon. But we’re not putting them through isokinetic dynamometry. We’re not re-imaging guys. I don’t use formal questionnaires with calf injuries…”* (Expert 18). While objective testing was valued for informing the RTP decision (Table 5), particularly instantaneous and repeated power capacities (Additional file 2: Table 3), experts also reported data should not be considered a panacea because between-side asymmetries were common to some extent even in healthy athletes, which could confound the situation.

**Table 4:** A clinical checklist to determine readiness to return to play after a calf muscle strain injury based on information provided by experts

Return to play criteria	✔or ✖
Symptom resolution and psychological readiness	
Self-reported symptoms: VAS 0/10 (pain, tightness, ‘cramping’ sensation)	☐
Self-perceived readiness & confidence to return to performance	☐
Residual clinical signs and impairments	
Palpation tenderness: VAS 0/10, length: 0 cm	☐
Weight bearing ankle dorsiflexion range of motion: normalised knee-to-wall lunge (cm) and straight leg stretch, asymmetries ≤ 10%	☐
Single leg calf raise test from the floor*: capacity (≥ 30 repetitions), asymmetry ≤ 10%	☐
Normalised strength-power qualities	
Loaded strength: sports-specific benchmark (knee extended, knee flexed)	☐
Power: normalised vertical and horizontal calf function; instantaneous and repeated tests (Supp file 2); asymmetries ≤ 10%	☐
Reconditioned for exposure to sport demands	
Running conditioning: total volume, volume across speed bandwidths, accelerations, decelerations	☐
Intensity of running and other dynamic activities: cutting, reactive agility, jumping, maximum velocity, maximum acceleration	☐
The mechanism of injury	☐
Successful re-integration into full training	
Return to full training for ≥ 1 session, pending the length of the rehabilitation period	☐
Consensus among stakeholders about readiness to perform at the required level (e.g. elite vs sub-elite vs amateur)	☐

*VAS* visual analogue scale, whereby ‘0’ represents no symptoms and ‘10’ represents the maximum of symptom severity. ✔ = achieved during rehabilitation, ✖ = not achieved and further rehabilitation may be required. *Note: testing single leg calf raise capacity from the floor (rather than a step) was perceived to limit the potentially significant impact of individual variation in ankle dorsiflexion range of motion

**Table 5:** Examples of screening and monitoring options for risk of future calf muscle strain injuries

Domain	Outcome of interest	Tests and example benchmarks
Non-modifiable intrinsic factors	Injury history	History of CMSI; other injury history or current sub-clinical state: foot (1st MTPJ, plantar fascia, bone stress, fractures), ankle (sprains, fractures, Achilles), knee, lumbar spine, other muscle strains (hamstring, quadriceps, adductor), including history of recurrent injuries; ethnicity
	Chronological age	Age: years
	Body mass index	kg m^2^
Range of motion and tissue extensibility	Weight bearing dorsiflexion Posterior extensibility	Knee-to-wall lunge test: Range of motion (cm); asymmetry ≤ 10% Standing flexion, or sit-and-reach test
Isolated calf strength	Foundation strength-endurance	Single leg calf raise test: capacity (repetitions to fatigue ≥ 30), asymmetry ≤ 10%, movement quality/ coordination. Metronome paced (30 beats/ minute); performed from the floor* to fatigue + / − a cut-off
	Loaded strength	Smith and/or seated calf machines: ideal relative strength benchmarks ≥ 1.0xBW for knee extended (0°) and ≥ 1.5xBW for knee flexed (90°); asymmetries ≤ 10%. Various RM regimes pending sport (1RM-8RM)
Power capacities and function during dynamic activities	Vertical	Single leg drop jump or single leg CMJ: total height (cm), reactive strength index, early RFD, late RFD, eccentric impulse; asymmetries ≤ 10%; movement quality/ coordination
	Horizontal	Forward hop test: distance (m); asymmetry ≤ 10%; movement quality/ coordination. Measured using a single or multiple hops ≥ 3, or as single-leg bound in high-level athletes; 20 m prowler sprint test
Exposure history	Recent	Details of any interruptions to usual exposure; details of recent program: velocities, volumes, exposure to specialised activities of the sport (multi-axial, acceleration, deceleration, metabolic), GPS data
	Medium–long term	Workload data; training age (i.e. number of years playing sport, completing on-field and off-field strength and conditioning activities/ exercises); GPS data; preseason completeness > 80%
	Loading conditions	Running surfaces; footwear; orthotics

*CMSI* calf muscle strain injuries; *MTPJ* metatarsophalangeal joint; *RM* repetition maximum; *RFD* rate of force development

^*^Note: testing single leg calf raise capacity from the floor (rather than a step) was perceived to limit the potentially significant impact of individual variation in ankle dorsiflexion range of motion

## Injury Prevention

### Aetiology and Risk Factors in Preventing ‘Calves’

**Fig. 6:**
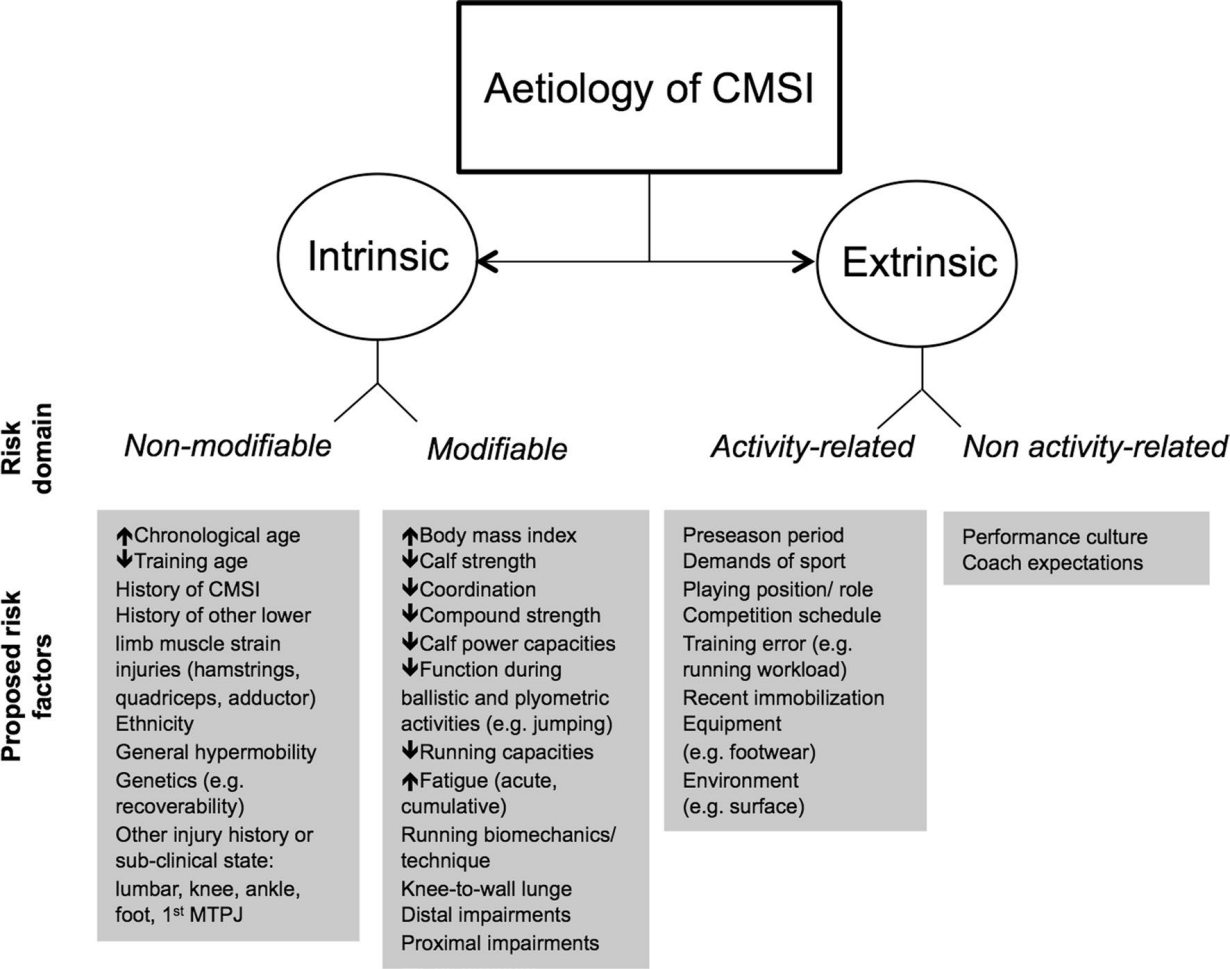
The aetiology of calf muscle strain injuries as proposed by 20 experts. *CMSI* calf muscle strain injuries, *MTPJ* metatarsophalangeal joint

Experts viewed identifying and synthesising information about the aetiology and risk factors of CMSI to be a key determinant of prevention. Experts focused primarily on the potential impact of intrinsic and extrinsic factors on the individual and their exposure (Fig. 6). This information was then used to guide decision-making about individualised exercise selection and load management.

### Do We Know Who Is at Risk of It Happening Again?

**Fig. 7:**
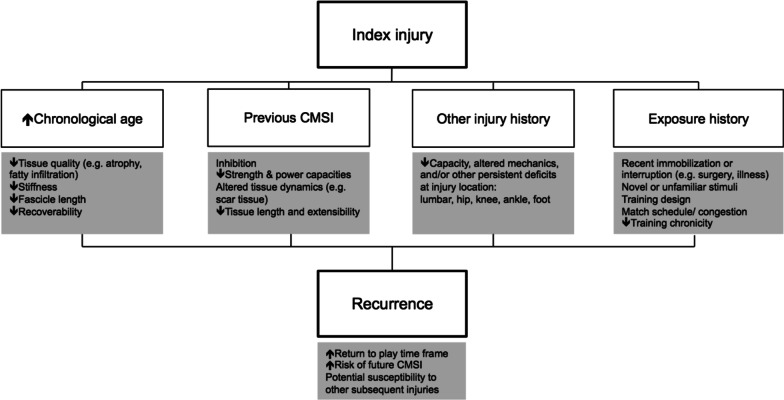
The potential mechanisms for how age, injury history and exposure increase susceptibility to recurrent calf muscle strain injuries. *CMSI* calf muscle strain injuries

A prevailing theme was the practical difficulty of recurrence prevention because they rarely occurred due to a single factor acting alone. Four factors were perceived to have the most significant impact on susceptibility to recurrence (Fig. 7). The mechanisms for how these factors may increase the risk of recurrent CMSI were explored based on information provided by experts.

### Can Risk Screening Tell Us Anything About ‘Calves’?

Experts routinely conducted preseason screening to generate a general risk profile for CMSI (Table 5); they did not expect these findings to strongly predict future CMSI. Baseline data were perceived to be most useful to provide normative scores for athlete monitoring and to design sports-specific prevention strategies. Where practical, optimal screening was individualised, based on historical, clinical (e.g. strength deficits related to a contraction mode and/or velocity) and performance data (e.g. instantaneous and/or repeated power capacities), and considered the impact of intrinsic factors (e.g. age and injury history) on calf function. Experts preferred to use ≤ 3 objective tests plus key subjective data: *“We’re going to get 10 or so new players in this year and have to screen them for the first time, and a player walks in, and I hate to be boring, but it would be injury history. Although we definitely do look at their movement because we have players who can’t even skip. They cannot two-footed skip for 30 s. I don’t know if I’d go straight to calf for that, but I’d like them to be able to skip. Can they jump rope for a sustained period of time? Can they do a simple movement?…If you can’t skip and we want you to run 14 k’s in a game, I’ve got a little bit of a mismatch there. We do things like calf rep max, body weight against the wall. I’d like to see people get to 30 on that. We metronome it.”* (Expert 15).

Most experts screened strength to gain an impression of general capacity/ load tolerance. While the single leg calf raise test had broad use as a measure of the foundation of calf strength-endurance (Table 5), many experts viewed loaded strength testing to provide a better indication of maximum capacity. Loaded strength tests were described to have the added utility of being able to be refined according to the sport (e.g. 1RM in rugby vs 6-8RM in Australian Football), as well as athlete impairments (e.g. inner range weakness; reduced eccentric strength). Favourable benchmarks were identified to guide athlete management across the sports canvassed (Table 5). Although some experts also raised the shortcomings of strength as a protective capacity against CMSI because it does not reflect the dynamic properties of the calf and provides diminishing value in the presence of compromised exposure. While power capacities during ballistic or plyometric tasks were measured to understand dynamic function, an emerging theme was that recording repeated measures (i.e. “*power-endurance*”, Expert 1) may better represent the ability to carry out work over the prolonged durations of most running-based sports. Repeated hopping, hopping after first performing a single leg countermovement jump, and single leg bounding were suggested methods to capture instantaneous (e.g. the first repetition) and repeated (e.g. subsequent repetitions) capacities.

### Athlete Monitoring to Prevent ‘Calves’

#### Mitigating the Risk of ‘Calves’ Once Training and Competition Begins

Experts monitored clinical data longitudinally to flag potential susceptibility to CMSI. Subjective (tightness, pain) and objective (stretch tolerance, single leg calf raise, hopping) tests were used to track fluctuations in calf capacity. Other injuries or sub-clinical states that could alter calf loading were also considered. Performance data were monitored during dynamic exercises (e.g. reactive strength index) and mined from GPS (e.g. maximum acceleration speed, total volume) databases to obtain a general risk profile for CMSI. Mitigation strategies were initiated if an elevated risk was detected, such as reducing exposure: *“It’s not just about exposing them, it’s about monitoring how they respond… sometimes they might have a bit of an adverse reaction to load. They might have some latent soreness after a heavy football session. But just be patient with them in those instances. Give them a session off and let them calm down before you go again. Whereas the guy who has never had a problem with his calf in the past, you can probably flog him in a footy session in the preseason, and if he gets calf soreness you can be more confident that he can probably push through the next football session with that soreness and not much will come of it. But maybe the guy that's got, you know the ‘genetic risk for soft tissue injury,’ he’s the one you can’t afford to do that with. So just be patient, be sensible, in those instances. But that’s where individualised modifications occur on the run a little bit,”* (Expert 18)*.* Common modifications included adjusting exposure to particular velocities, the number of accelerations and decelerations, and cutting: *“If we felt that there’d been a real spike in their exposure through the game, which is a non-modifiable factor really, in training the following week potentially when they have some ‘small-sided games’ or other drills that are very ‘in-tight’ and close with high change of direction units, we might modify them out of those just to mitigate that risk,”* (Expert 16). Total volume was highlighted for offsetting the risk of gradual-onset CMSI involving soleus, which were attributed to cumulative overload, whereas gastrocnemius injuries were perceived to be more sensitive to high-intensity activities such as jumping. If experts detected an elevated risk, exercise selection could be adjusted to avoid compounding the situation, particularly ballistic exercises and heavy calf strengthening. A history of *limited* exposure could also increase susceptibility to CMSI, with three primary flags identified based on the responses: (1) a reduced training age; (2) an illness, injury, or external factors recently interrupting loading; and (3) resumption following a break (e.g. the off-season).

**Fig. 8:**
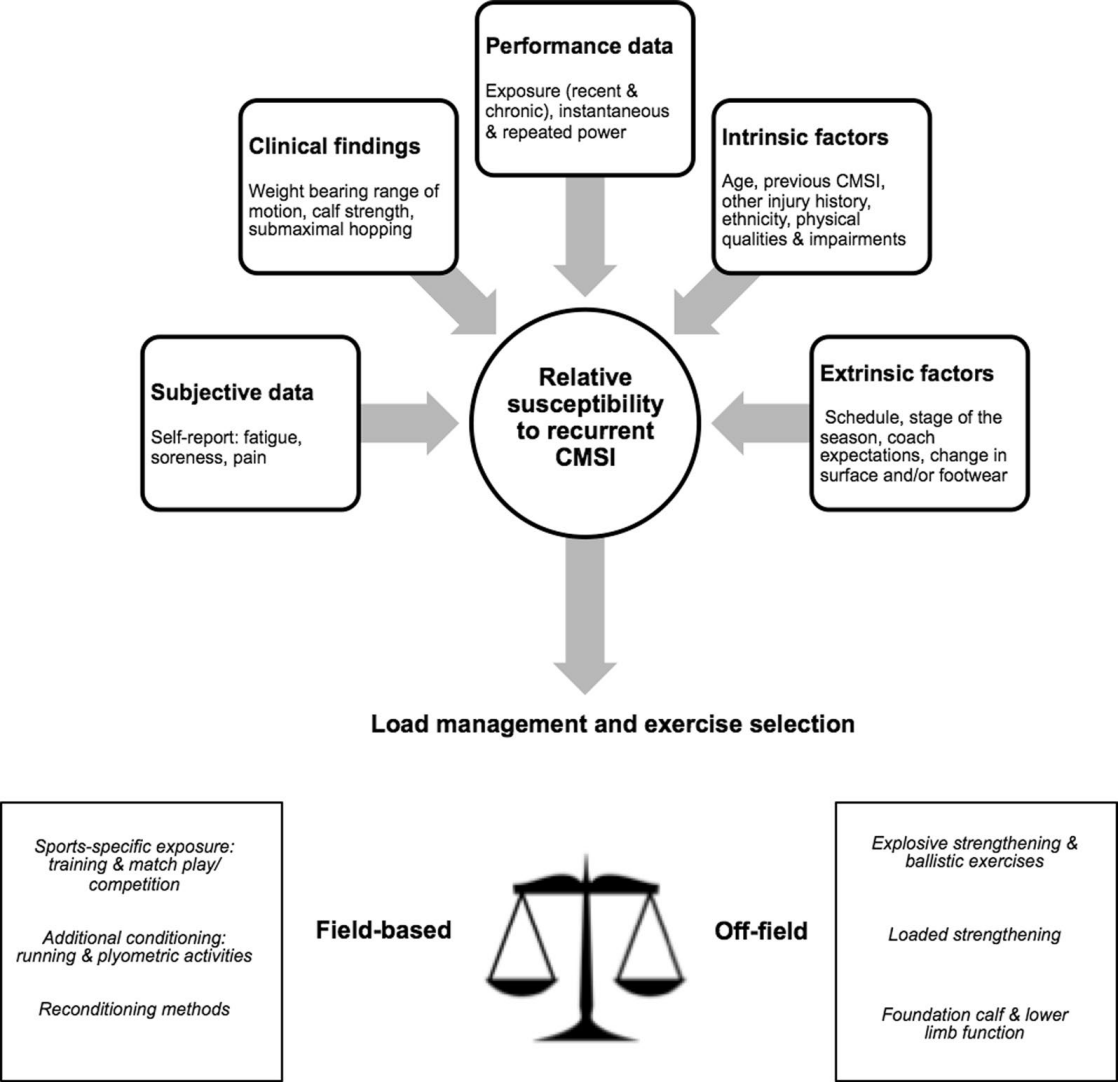
Evaluating and managing susceptibility to recurrent calf muscle strain injuries

Experts described athlete monitoring to underpin optimal management after CMSI as well (Fig. 1). For example, experts staged early progress by comparing clinical asterisk signs (e.g. palpation tenderness, strength, range of motion) with initial examination findings. Later, these tests were employed by experts to detect adverse reactions to load and guide the rate of functional progression. A key concept was to adopt a monitoring approach rather than progressing to symptom provocation during exercises and/or running because sensory feedback was not always a reliable indication of tissue integrity after CMSI. Persisting impairments such as weakness or inhibition were also perceived to be more likely if progressions occurred at the expense of symptoms or movement quality, predisposing to recurrent CMSI: *“One of the things we see when we load it, is that the manner in which they get the contraction can vary greatly. So while we try to have an external load goal, you can have certain athletes lift that amount and be very different in the way that they do it. So we also look for good quality in the movement,”* (Expert 7). Similarly, while pain-threshold running reportedly had utility for other muscle strains, which were by nature “*more self-limiting*,” (Expert 18), CMSI showed different symptomatology and running-related symptoms could show acute susceptibility or that a recurrence had occurred. Running frequency was another important component to monitor while building intensity and volume: *“Frequency of running sessions is probably the thing that breaks them the most. Like when they first start to do back to back days,”* (Expert 1) For running-related CMSI, experts utilised GPS data to monitor exposure to the mechanism of injury: *“One thing that always does amaze me at times is just how specific muscle strain injuries can be to the mechanism of injury… whether that’s a sort of a very specific, localised tissue issue, or whether it’s a bit of fear and apprehension, or a bit of both, who knows,”* (Expert 18). After RTP, exposure to running activities with the largest calf demands (*“The two key ones that probably fit for the calf is our moderate speed running, and probably the ‘accel-decels’,”* (Expert 11) and exercise selection were monitored to prevent subsequent injury (Fig. 1): *“It might take another month after they have returned to play before they are back up to normal loads. If you don’t want a recurrence, protect their workload even after they have returned to play…keeping them ‘off legs’ an extra day, and maybe protecting them on another, so they have recovery between major sessions and games, is really important,”* (Expert 1). A model to estimate susceptibility to recurrent CMSI was created from information provided by experts (Fig. 8). Using this information to guide athlete management in real-time by balancing load was especially critical for athletes with risk factors for CMSI.

### Here’s an Idea: Why Don’t We Just Stop ‘Calves’ from Happening Instead?

**Fig. 9:**
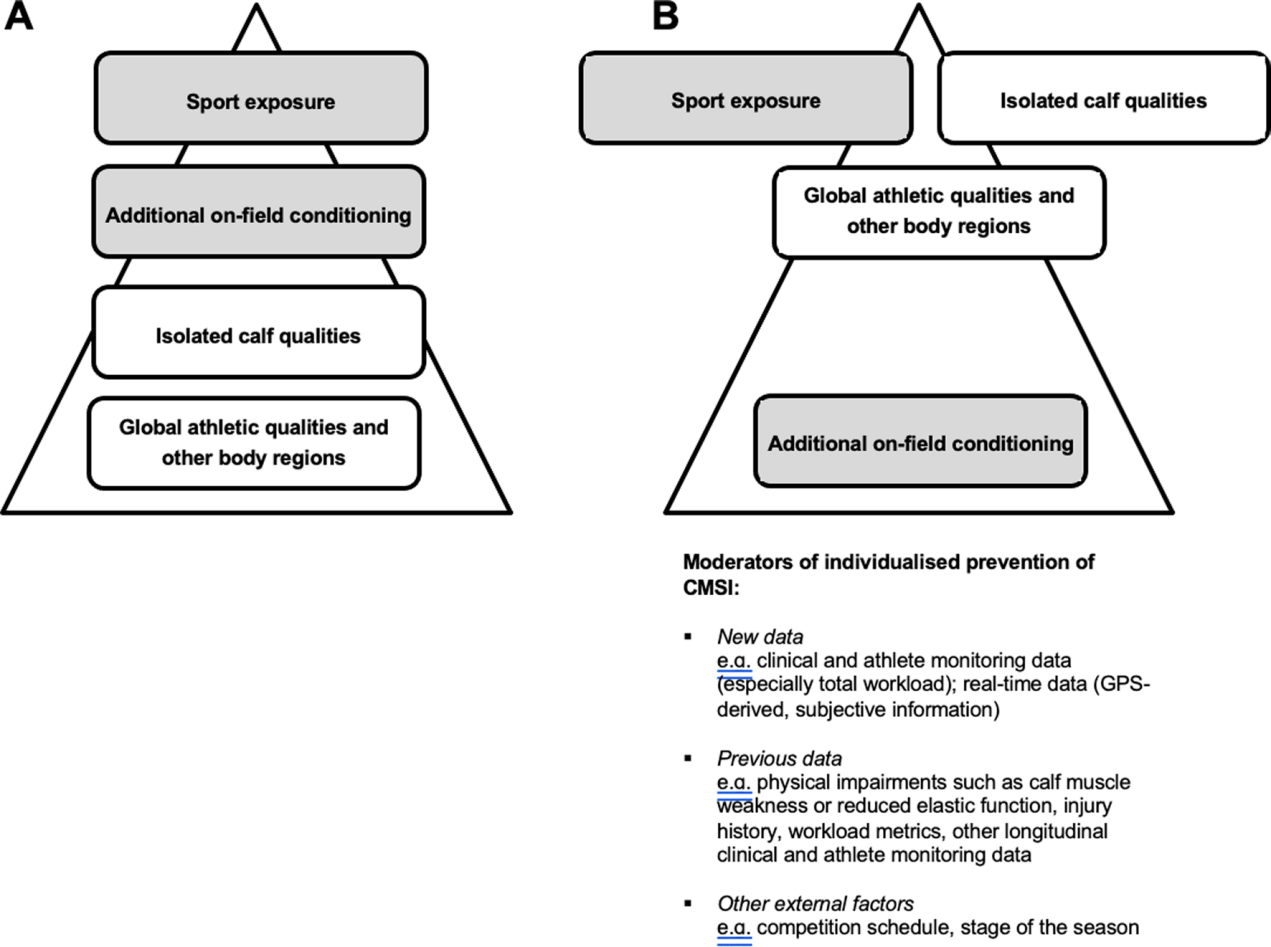
**A** A typical hierarchy of implementation for prevention strategies for CMSI in healthy elite athletes from running-based sports. **B** An example of an adjusted hierarchy for an older athlete with a history of calf muscle strain injuries. Legend: Shaded boxes represent on-field activities/ focuses of injury prevention; white boxes represent off-field activities/ focuses of injury prevention

A universal injury prevention program was not identified for CMSI due to the diversity in the demands on the calf between sports: *“In a team sport there are a lot more elements that you are preparing for. In a middle distance runner you are preparing to run, and that’s it, and that’s all you do,”* (Expert 16)*.* To begin the prevention process some advocated first conducting a ‘*needs analysis’* to identify the capacities required and potential aetiologies of CMSI in the sport, including the likely injury mechanisms and muscle injured. For these experts, this information underpinned the focus of screening and athlete monitoring, and guided the implementation of preventative exercise selection and load management. A hierarchy of implementation was created from information provided by experts about the prevention priorities in running-based sports (Fig. 9A). Overall, chronic and uninterrupted exposure to the sport and the specific activities involved in that sport were considered the most important strategies for resilience to CMSI. However, specific prevention priorities for a particular athlete were subject to change based on the athlete’s intrinsic factors and other relevant information (e.g. athlete monitoring). Figure 9B shows a theoretical example of an adjusted hierarchy reflecting an individualised approach for an older athlete with a history of CMSI. In this example, sport exposure and developing intrinsic calf qualities are equally important for preventing CMSI (Fig. 9B).

Uninterrupted sports exposure was most salient (Fig. 7A). *“If you get them through preseason, where the program is well-structured, and they get good exposure to football, they get good exposure to running, it has consistent week-to-week progressions, you aren’t doing dumb jumps in week-to-week loading. You know, just well-crafted, common sense; you will get 95% of your guys through without calf injuries ever being a major issue for you,”* (Expert 18). Exposure was described to protect against CMSI by preparing the calf MTU to the specific work demands of the sport—an important point because the relative load and loading rates during dynamic activities are not reproducible using conventional off-field exercises. Excessive or poorly timed exposure could also increase the risk of CMSI, irrespective of how well the other components are designed. Manipulating load exposure was encouraged when athletes who are older or have a history of CMSI and/or other potential risk factors show evidence of being under-recovered. Additional on-field conditioning built resilience to CMSI, particularly during the preseason, which focused on exposure or technique during acceleration, velocity, cutting and agility activities, as well as fitness. Plyometrics were recognised to provide a large protective benefit for the calf. Experts identified these exercises elicit adaptations associated with improving the elastic function of the MTU, with the added benefit of reducing cumulative work by shortening ground contact times.

Calf strengthening was described to be a cornerstone of building muscle capacity and resilience to CMSI: *“The trouble with calf strength is it is like a little magic thing that disappears on you. You haven’t done something, and you try to, especially in older men, and all of a sudden you can only do 5 calf raises. But you’ve been running and you’ve been doing all of this other activity,”* (Expert 12). In sprinters and rugby athletes, ≥ 2 × bodyweight (BW) was considered to be a minimum level of strength to protect against CMSI. Australian Football (1.0–1.5 × BW) and soccer (0.8–1.0 × BW) had lower benchmarks and greater consideration of strength-endurance (e.g. 8RM). Substantial differences in strength requirements between playing positions were also reported. For example, rugby front rowers required strength ≥ 2 × BW, and very high eccentric strength, whereas fullbacks needed a greater degree of both explosive and strength-endurance. The single leg calf raise also had a universal role for training foundation strength-endurance and motor control, especially in athletes with a low training age or gross weakness. At least 30 repetitions to fatigue and symmetry (asymmetry ≤ 10%) were expected: *“You probably do need a minimum level of calf capacity. As I said, our calf testing hasn’t been predictive in terms of the guys that can bang out 35 reps haven’t necessarily been immune. So I think it’s not necessarily a predictive marker, but having said that I think a guy that can only do 15 you just have to logically assume that capacity is going to be an issue for them,”* (Expert 16)*.* Some experts criticised relying on conventional strength training to prevent CMSI because it does not address the velocities and loading rates required during dynamic activities. Advanced exercises were used to provide a protective benefit in these areas, such as explosive resistance training and resisted locomotion (e.g. sled, prowler).

## Discussion

This qualitative study explored best practice for the assessment, management and prevention of CMSI. Our findings represent the perspectives and experiences of 20 experts practicing in 9 countries, across a range of sports. These data have enabled practical information to be developed with respect to evaluating injury characteristics, rehabilitation, RTP decision-making and prevention strategies.

### Start ‘Big’ by Understanding the Epidemiology Because (Spoiler) this Information is the Basis for Everything That Follows

Epidemiology was a critical first consideration for experts to frame clinical reasoning in the assessment, management and prevention of CMSI, since these features impact exposure to aetiological factors and the potential injury mechanisms encountered [14]. For example, experts highlighted rugby players were more commonly afflicted by CMSI involving gastrocnemius due to the running demands of the sport and exposure to specific injury mechanisms. This information subsequently directed the assessment, rehabilitation and development of prevention strategies for these athletes. Epidemiological information could also be helpful for identifying the likely athlete intrinsic characteristics, such as their somatotype, athletic traits, age and injury history [14]. From an injury prevention perspective, effective implementation is likely impossible without an appreciation of these factors together with sports-specific aetiological inputs and injury mechanisms [15]. A similar phenomenon exists with respect to injury management [40].

### Next, Make It ‘Small’: Rigor in the Examination Simplifies What can Seem a Complex Injury Situation

Experts safeguarded accuracy during clinical-decision making by utilising a systematic approach to examining the injured athlete, the pathology and identifying important contextual factors. The subjective examination served to obtain precise information about symptomatology and the inciting event (if any) [40], underpinning data-gathering during the objective examination to confirm the diagnosis and severity. The muscle injured, mechanism of injury, injury type, functional impairment, severity of disruption on MRI and rate of recovery were all considered by experts to accurately estimate prognosis after CMSI. To obtain the most comprehensive impression, both broad and specific injury characteristics should be considered in an integrative fashion with the specific attributes of the athlete and the sport they are participating in kept firmly in mind [14, 15]. Rigor early also provided the foundation from which other clinical areas could be explored, such as considering how multiple risk and predisposing factors interact in each injury situation [17]—which was used to shape rehabilitation and the RTP decision.

### Is the Pathology or Functional Progression Foremost in Guiding Injury Management?

Regardless of the pathology, therapeutic loading and restoring calf capacity were early priorities, and functional progression guided progress as soon as experts had the opportunity to commence loading. Data from recent studies support this concept—early loading of CMSI results in faster recovery [41] and may improve pain and confidence [42], irrespective of injury characteristics such as the muscle involved, anatomical location of injury, and tissue type injured. Despite the recent shift away from managing muscle strains according to the estimated time taken for the underlying pathology to resolve, as recommended by Hickey et al. for hamstring strain injuries [43], experts did highlight situations where pathology was believed to be an important consideration for selecting exercises and planning functional progression. *‘Problem calves’* were one example that required even greater time devoted to building load tolerance and exposure to activities involving high loading rates. Balancing an appreciation of the pathology while progressing at the fastest rate possible appears to be fundamental to optimal injury management in elite sport. This concept is supported by previous research into hamstring and groin injuries [44–46].

### Ok, Great. We have a Recipe Now—But There is More Than One Way to Skin a Cat

Optimal management in our sample was perceived to involve six phases and a dynamic rehabilitation sequence, which is refined by the needs of the sport, the individual and the pathology. While experts recognised the need for a systematic approach to manage CMSI, citing past failures and experiences as the impetus, vast differences in what is considered *‘optimal’* may be expected between sports and between athletes. Field-based activities and gym-based exercises were refined to reflect the demands at RTP, and were further moulded by individual (e.g. age, injury history, physical impairments) and injury (e.g. muscle injured, severity, mechanism of injury) characteristics. A novel concept was using ‘reconditioning’ to better prepare the calf MTU for dynamic activities. While training studies have shown resisted locomotion may benefit acceleration and sprinting speed [47, 48], further exploration is warranted to determine its role after CMSI.

### Don’t Run a Calf Like you Would Run a Hamstring (or a Quad, or an Adductor)!

Experts acknowledged criteria for running after CMSI is more stringent relative to other types of muscle strains [49–51], because running is a high-load scenario for the calf even at the slow speeds prescribed initially [39]. Most experts accepted a slightly delayed return to running in order to reduce the likelihood of early recurrence [16]. Data from two recent studies support this approach. Running early (≤ 4 days) following lower limb muscle strains in 70 Australian Football players, of which ≈25% were CMSI, was associated with an elevated risk of subsequent injury after RTP [52]. A slight delay, however, may result in a lower risk, without negatively impacting rehabilitation time frames [53]. Progressing running after CMSI presented unique constraints as well. A graded approach was needed to effectively recondition the calf to all running-based activities, especially soleus which faces the greatest work demands [39, 54–56]. For athletes with large running workloads, total volume was the final milestone due to the potential susceptibility to recurrence once fatigued. This was perceived to be a different situation to other muscle strains, which are often more sensitive to increasing the intensity of activities (e.g. hamstrings: high-speed running [57, 58]; adductors: cutting [59]).

### Art Meets Science in Return to Play Decision-Making

Investigations into muscle strains have focused on the predictive value of clinical and radiological factors on the time taken to RTP and recurrence, showing mixed evidence across the hamstrings [60–62], adductors [63–65] and calf [7, 66, 67]. Baseline clinical and radiological information may together help to estimate recovery after CMSI, whereas clinical factors best inform the risk of recurrence [7, 8, 31]. This information may help guide the rate of functional progression and ensure subsequent injury risk is minimised. Further research is needed to validate how progress is staged between injury onset and RTP, such as clinical [46, 68] and performance [52, 57, 58] data related to CMSI, as well as outcome measures that account for the diversity in sport demands. Consistent with our study, repeating the MRI to confirm healing does not optimise the RTP decision [69]. Combining information from a variety of sources allows stakeholders to reach an optimal RTP decision and facilitate performance, which is supported by a recent expert consensus [70].

### There is Hope for Preventing CMSI. (*Disclaimer: don't Expect Single Interventions to Do the Trick)

For prevention strategies to be effective, contributing factors must first be recognised, which include epidemiology, risk factors, mechanical considerations, and the environment [16, 40, 71]. Susceptible athletes required even more individualised attention, such as those who have a history of CMSI or impairments that reduce load-tolerance. Consistent with previous research, we did not find a “one-size fits all” method for translating information about injury aetiology to designing a universal prevention program. While causation and risk factors are important to identify, more meaningful information may be gained from identifying changes in the risk profile over time because responses to exposure and relative load tolerance can be unpredictable due to the numerous factors at play [40, 72]. This highlighted the largest barrier to implementing traditional prevention strategies for CMSI: “protective” calf qualities undergo fluctuations. Practice has shifted from using screening tests such as the single leg calf raise with the expectation that subsequent CMSI can be predicted [73, 74]. Objective data are best applied together with subjective information about injury and exposure histories, providing an estimate of the risk profile for CMSI. This approach permitted individualised prevention using load management and exercise selection strategies [73], while affording consideration of the multitude of factors that impact an athlete’s risk profile (e.g. behavioural qualities, training design, individual skill, coach expectations/club culture and environmental factors [40, 75]).

### Recurrence Prevention is more About What you Do: Simply Taking Longer is not Always Protective

Preventing recurrent CMSI can be challenging due to their unpredictability. Experts did not find simply extending the rehabilitation period to be an effective safeguard to avoid recurrence. In support of this point, a recent study found no association between the precise length of the rehabilitation period and the risk of recurrent CMSI [31]. Delaying RTP may also increase the risk of a subsequent injury if it compromises exposure to high-load activities [52, 53]. Other factors such as older age and injury history [31], deficits in strength and plyometric function, and exposure history, may be more influential on risk of recurrence. In particular, susceptible athletes were perceived to have persisting impairments that reduce tolerance to high-load activities such as running [76]. Experts highlighted practical methods and considerations to restore optimal function and mitigate the risk of recurrence, including *‘problem calves’*. Athlete monitoring was a major strategy to ensure comprehensive management and reduce the likelihood of having persisting impairments at RTP that may predispose to recurrence, as previously shown for the hamstrings [60, 68, 77]. Identifying persisting impairments [60, 78, 79] may be a way to determine *‘at-risk’* athletes and inform immediate decisions relating to readiness to RTP, as well as exercise selection to address these modifiable impairments [80] or improve structural integrity at locations vulnerable to CMSI [81, 82]. Monitoring athlete status rigorously is important because > 50% of recurrent CMSI occur during rehabilitation or soon after RTP [7, 31].

Ongoing athlete management strategies aided the prevention of subsequent injury after RTP as well. Preventing recurrent CMSI may require prolonged attention because athletes are susceptible for longer than other muscle strains (≈4 months) [8] and recurrences can cause prolonged time-loss [7, 83]. After RTP, athletes are also susceptible to other injuries [8, 9] and this elevated risk may not resolve for ≈3 months [84]. It is unknown whether this is due to the impact of pathology associated with the CMSI or altered exposure, but experts used rehabilitation as an opportunity to identify risk factors and impairments relevant to subsequent injury risk. These factors were considered in exercise selection, staging progress and RTP clearance. While the length of the surveillance window post-RTP appears to vary due to the impact of intrinsic (age; previous CMSI; other injury history) [8, 31, 85] and extrinsic (stage of the season; playing position) [86, 87] factors, as well as the pathology (muscle involved; index versus recurrent injury [7]), monitoring exposure for ≥ 2 months is likely critical to the ongoing success of managing CMSI [52, 53].

## Strengths and Limitations

To reduce potential bias associated with geography and the field of practice, this study involved expert researchers and/or clinicians working at the elite level of competition, spanning a range of sports, from around the world. The collective expertise of the participants was represented by the range of clinical roles, postgraduate qualifications and relevant research fields, which created breadth in theoretical knowledge as well. The qualitative interview study design and analysis permitted in-depth exploration of complex concepts and clinical-reasoning, which may be impractical using a quantitative approach or even a single qualitative survey approach. The qualitative design may result in potential biases inherently, such as interviewer bias, as well as the potential risk of bias associated with the author team being made up of a group of clinician-researchers. Given participants had diverse clinical roles and backgrounds, each participant did not necessarily have expertise in all of the areas discussed. Participants were also required to speak the English language, potentially limiting data sources.

## Conclusion

Experts optimised clinical reasoning at the time of injury onset by using a structured approach for injury diagnosis and estimating prognosis. Best management after CMSI was perceived to involve transitioning the athlete through six phases extending beyond the RTP date, each embedded with principles to guide the clinician. The final RTP decision was encouraged to be consensus-driven and informed by clinical and athlete monitoring data. While a universal prevention program may not be viable due to diversity in calf demands between sports, a multifaceted approach involving individualised load management and exercise selection could provide the best preventative effect.

## Supplementary Information

**Additional file 1.** Interview Schedule of Questions.

**Additional file 2.** Further details of the initial clinical examination, sings of poor calf function during the calf raise test, and objective testing options for power qualities at RTP.

## Data Availability

Complete transcripts of interview data are not available, but some excerpts may be available upon reasonable requests.

## Abbreviations

AFLPTA: Australian Football League Physiotherapists Association
CMSI: Calf muscle strain injuries
DOMS: Delayed onset muscle soreness
FDL: Flexor digitorum longus
FHL: Flexor hallucis longus
GPS: Global positioning system
MOI: Mechanism of injury
MRI: Magnetic resonance imaging
MTPJ: Metatarsophalangeal joint
MTU: Muscle–tendon unit
NWB: Non weight bearing
RM: Repetition maximum
RTP: Return to play
SSC: Stretch shortening cycle
VAS: Visual analogue scale
WB: Weight bearing
