# Supplementary material for: The Assessment, Management and Prevention of Calf Muscle Strain Injuries: A Qualitative Study of the Practices and Perspectives of 20 Expert Sports Clinicians
Source: Sports Med Open. 2022 Jan 15;8:10. doi: 10.1186/s40798-021-00364-0 (PMC8761182; doi:10.1186/s40798-021-00364-0)
Supplement: Supplementary file 1 — Additional file 1. Interview Schedule of Questions. [file 40798_2021_364_MOESM1_ESM.pdf]

The assessment, management and prevention of calf muscle strain injuries: A qualitative study of the practices and perspectives of 20 expert sports clinicians

Sports Medicine - Open

Brady Green[1], Jodie A McClelland[1], Adam I Semciw[1,2], Anthony G Schache[1], Alan McCall[3,4], and Tania Pizzari[1]

[1]La Trobe Sport and Exercise Medicine Research Centre, La Trobe University, Melbourne, Australia
[2]Northern Centre for Health Education and Research, Northern Health, Victoria, Australia
[3]Arsenal Performance and Research Team, Arsenal Football Club, the United Kingdom
[4]School of Applied Sciences, Edinburgh-Napier University, United Kingdom

Corresponding author details:

Mr Brady Green
La Trobe Sport and Exercise Medicine Research Centre
La Trobe University, Melbourne, Australia
E: B.Green2@latrobe.edu.au
ORCID: https://orcid.org/0000-0003-1135-0033

LA TROBE UNIVERSITY

COLLEGE OF SCIENCE, HEALTH & ENGINEERING
SCHOOL OF ALLIED HEALTH

Mailing address
La Trobe University
Victoria 3086 Australia

T + 61 3 9479 5815
E: asklatrobe@latrobe.edu.au
www.latrobe.edu.au/health

MELBOURNE CAMPUSES
Bundoora
Collins Street CBD
Franklin Street CBD

REGIONAL CAMPUSES
Bendigo
Albury-Wodonga
Mildura
Shepparton

Interview Schedule of Questions (HEC18060)

The interview will broadly cover the diagnosis, management, and prevention of calf strain injuries in athletes. The following schedule of questions is intended as a **guide** only, to ensure that all relevant areas are covered by the interviewer.

- Take me through how you would make the diagnosis of a calf muscle injury.
  - Subjective:
    - Mechanism / history of presenting condition
    - Past history
    - Aggravating factors
  - Examination
  - Investigations
  - Grade of the injury
  - Differential diagnosis

- Take me through your current management of a calf muscle injury (for each grade).
  - Acute management
    - Passive: manual techniques
    - Active: early exercises
  - Post acute
    - Passive: manual techniques
    - Active: exercises
    - Areas most important to address during rehab
- Return to activity, including running program
    - Early running program
    - Approach to progressing running (volume, intensity)

- What criteria do you use to identify readiness to return to sport?
- What techniques do you use in an attempt to prevent subsequent calf muscle injury?
- Can anything be done to prevent first time calf muscle injuries? (Current screening and monitoring that may be useful)
- Anecdotally, have you identified any predictors for calf muscle injuries?
