# Supplementary material for: The Assessment, Management and Prevention of Calf Muscle Strain Injuries: A Qualitative Study of the Practices and Perspectives of 20 Expert Sports Clinicians
Source: Sports Med Open. 2022 Jan 15;8:10. doi: 10.1186/s40798-021-00364-0 (PMC8761182; doi:10.1186/s40798-021-00364-0)
Supplement: Supplementary file 2 — Additional file 2. Further details of the initial clinical examination, sings of poor calf function during the calf raise test, and objective testing options for power qualities at RTP. [file 40798_2021_364_MOESM2_ESM.docx]

**The assessment, management and prevention of calf muscle strain injuries: A qualitative study of the practices and perspectives of 20 expert sports clinicians**

**Sports Medicine - Open**

Brady Green^1^, Jodie A McClelland^1^, Adam I Semciw^1,2^, Anthony G Schache^1^, Alan McCall^3,4^, and Tania Pizzari^1^

^1^La Trobe Sport and Exercise Medicine Research Centre, La Trobe University, Melbourne, Australia

^2^Northern Centre for Health Education and Research, Northern Health, Victoria, Australia

^3^Arsenal Performance and Research Team, Arsenal Football Club, the United Kingdom

^4^School of Applied Sciences, Edinburgh-Napier University, United Kingdom

**Corresponding author details:**

Mr Brady Green
La Trobe Sport and Exercise Medicine Research Centre

La Trobe University, Melbourne, Australia

E: B.Green2@latrobe.edu.au

ORCID: https://orcid.org/0000-0003-1135-0033

**Appendix 2**

Table 1. *Initial subjective examination*

| **Domain:** | **Key areas:** | **Baseline outcomes:** |
| --- | --- | --- |
| 1. The presenting injury | i. History of onset | The presence (yes/no) of an inciting incident. If yes, the mechanism of injury  The duration of symptom onset: immediate; cumulative; days |
|  | ii. Self-reported symptoms | Symptom qualities and intensity: pain; unresolved ‘tightness’ or ‘cramping’; paraesthesia; numbness  Location(s) of symptoms: focal; diffuse  Perceived level of severity or functional impairment |
| 2. The injured athlete | i. Intrinsic factors | Non-modifiable factors: history of CMSI; chronological age; training age; ethnicity; other injury history, including recurrences (foot, ankle, knee, spine, hamstring, quadriceps, adductor)  Modifiable factors: mobility; strength-power capacities; fitness/ conditioning to running and ballistic activities |
|  | ii. Other predisposing factors | Recent period of relative off-loading or immobilization (e.g. illness, surgery, injury); monitoring or ‘wellness’ flags: fatigue, recovery, sleep, illness; impairments or functional restrictions related to current clinical/ sub-clinical state: 1^st^ MTPJ, plantar fascia, ankle, Achilles, knee, bone stress, spinal. |
| 3. The injury context | i. Extrinsic factors | Demands of the sport: playing position, playing style, stage of the season; recent and long term history of training and competition |
|  | ii. Other contextual factors | Change in footwear and/or orthotics  Potential for training error: large changes in exposure or unaccustomed stimuli (e.g. running surface; eccentric exercise) |

**Legend:** CMSI= calf muscle strain injuries, MTPJ= metatarsophalangeal joint

Table 2. *Initial objective examination*

| **Domain:** | **Key areas:** | **Baseline outcomes:** |
| --- | --- | --- |
| 1. Observation | i. Local observation    ii. Other structures | Relative calf bulk (size, shape); observable signs of severe CMSI; observable signs of other injury (e.g. contusion, Achilles rupture or tendinopathy)  Relative muscle bulk (size, shape) compared to contralateral limb: posterior gluteus maximus, hamstrings, quadriceps |
| 2. Palpation | i. Tenderness | Discrete location if discernable: gastrocnemius (medial, lateral), soleus, Achilles; pain; maximum length of tenderness (cm); symptom quality |
|  | ii. Tactile qualities | Focal spasm; palpable defect or deformity: Achilles tendon, medial gastrocnemius, superficial triceps surae confluence; evidence of direct injury where relevant (e.g. contusion) |
| 3. Stretch tolerance | i. Passive dorsiflexion: KF, KE | Non weightbearing range of motion (°); pain; other symptoms; symptom quality |
|  | ii. Knee-to-wall lunge | Weightbearing range of motion and asymmetry (cm); pain; other symptoms; symptom quality |
|  | iii. Straight leg stretch at wall | Weightbearing range of motion asymmetry (cm); pain; other symptoms; symptom quality |
| 4. Isolated function | i. Isometric contraction: KF, KE | Non-weightbearing plantar flexion: capacity, pain, other symptoms. |
|  | ii. Single leg calf raise: KF, KE | Weightbearing plantar flexion: capacity, pain; other symptoms; symptom quality; antalgic strategy  Graded, as appropriate: a) double leg, b) double leg concentric, single leg eccentric, c) SLCR |
| 5. Dynamic capacity | i. Plyometric function | Capacity; pain; other symptoms; symptom quality; antalgic strategy Graded, as appropriate for jumping and hopping: a) double leg vertical, b) single leg vertical, c) single leg horizontal |
|  | ii. Locomotive activities | Capacity; pain; other symptoms; symptom quality; antalgic strategy Graded, as appropriate: a) walking, b) submaximal jogging, c) linear run through, d) cutting and change of direction, e) sprinting, f) maximum acceleration from inert position |

**Legend:** *pain= (1) presence (yes/no) and (2) extent (VAS:x/10); KF= knee position in flexion; KE= knee position in extension; cm= centimetres; °= degrees measured using goniometer; capacity= the ability to perform the task (yes/no); other symptoms= ‘tightness’, ‘cramping’, or neural symptoms elicited; symptom quality= focal versus diffuse; SLCR= single leg calf raise; antalgic strategy= observable presence of antalgic strategy (yes/no)

Figure 2. *The sickle sign during the single leg calf raise*


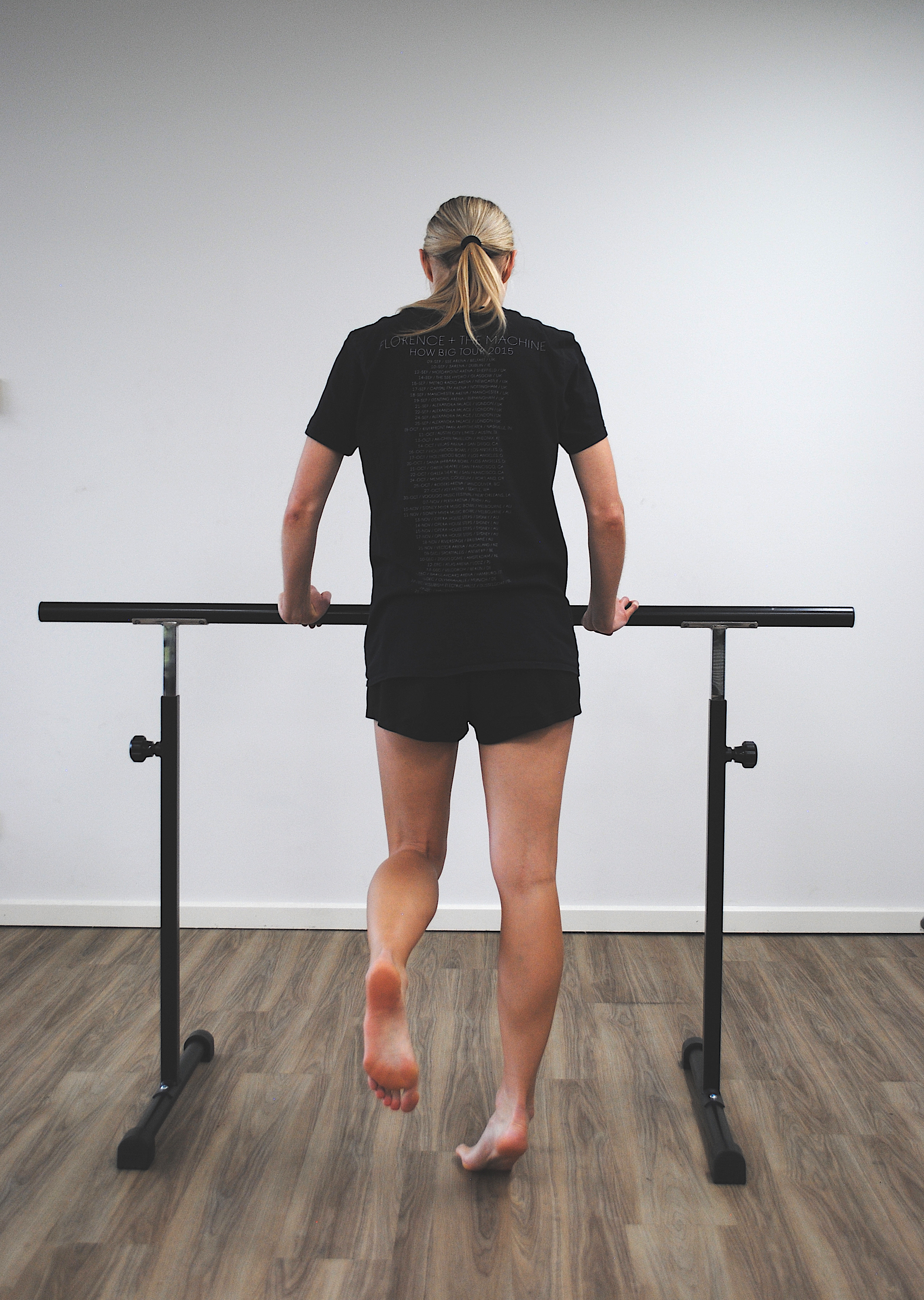

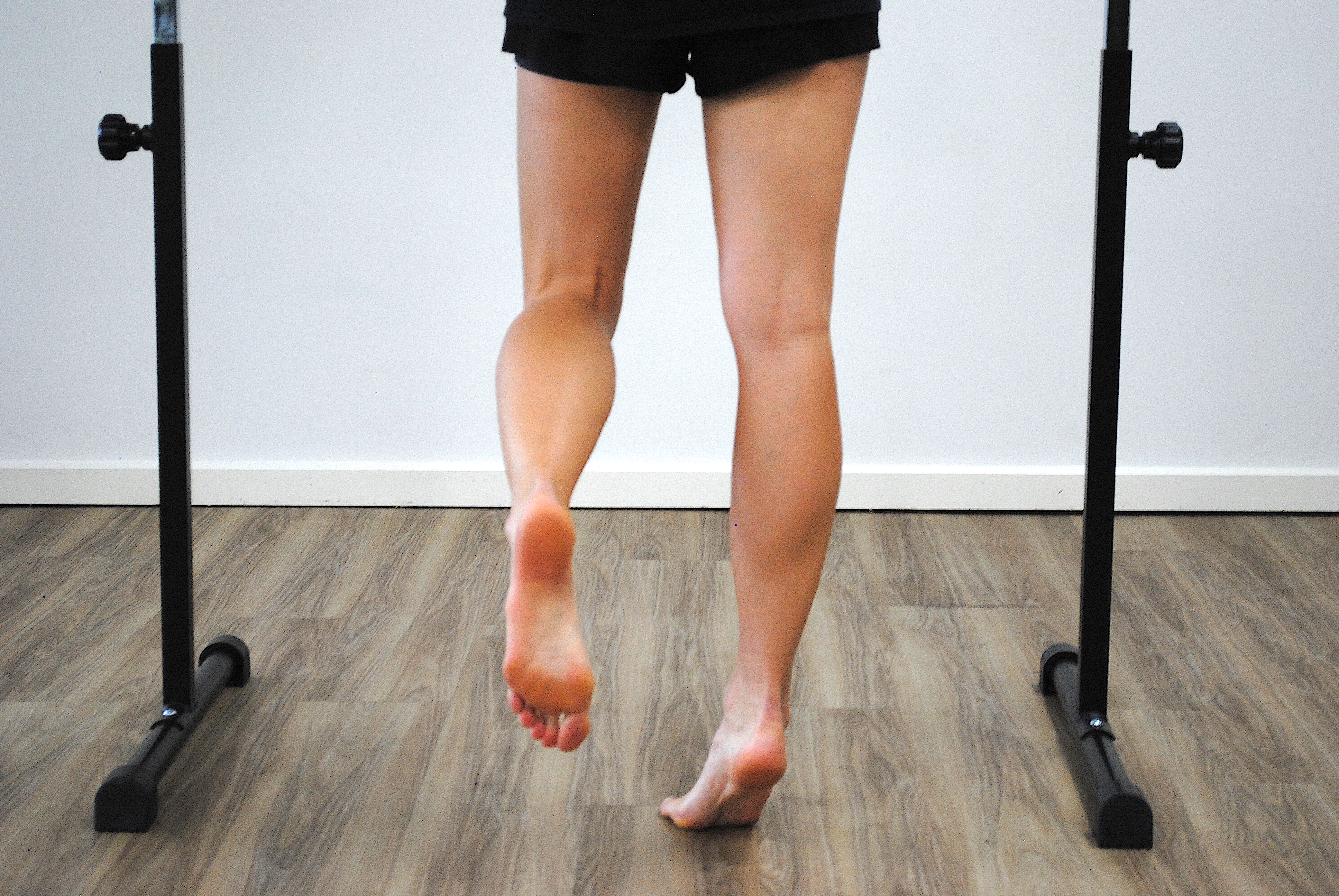


Figure 3. *Toe-clawing during the bent knee calf raise*

*
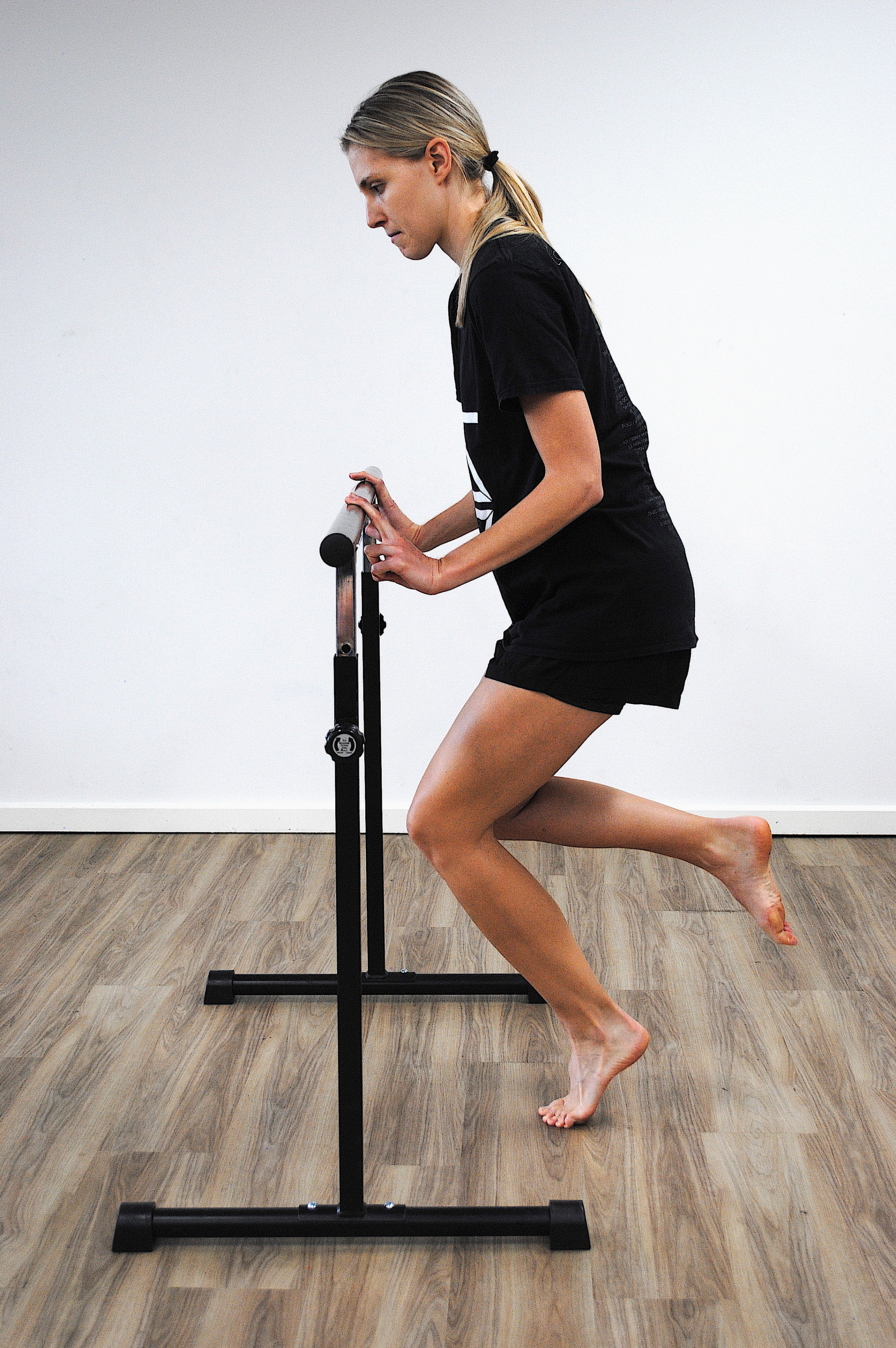

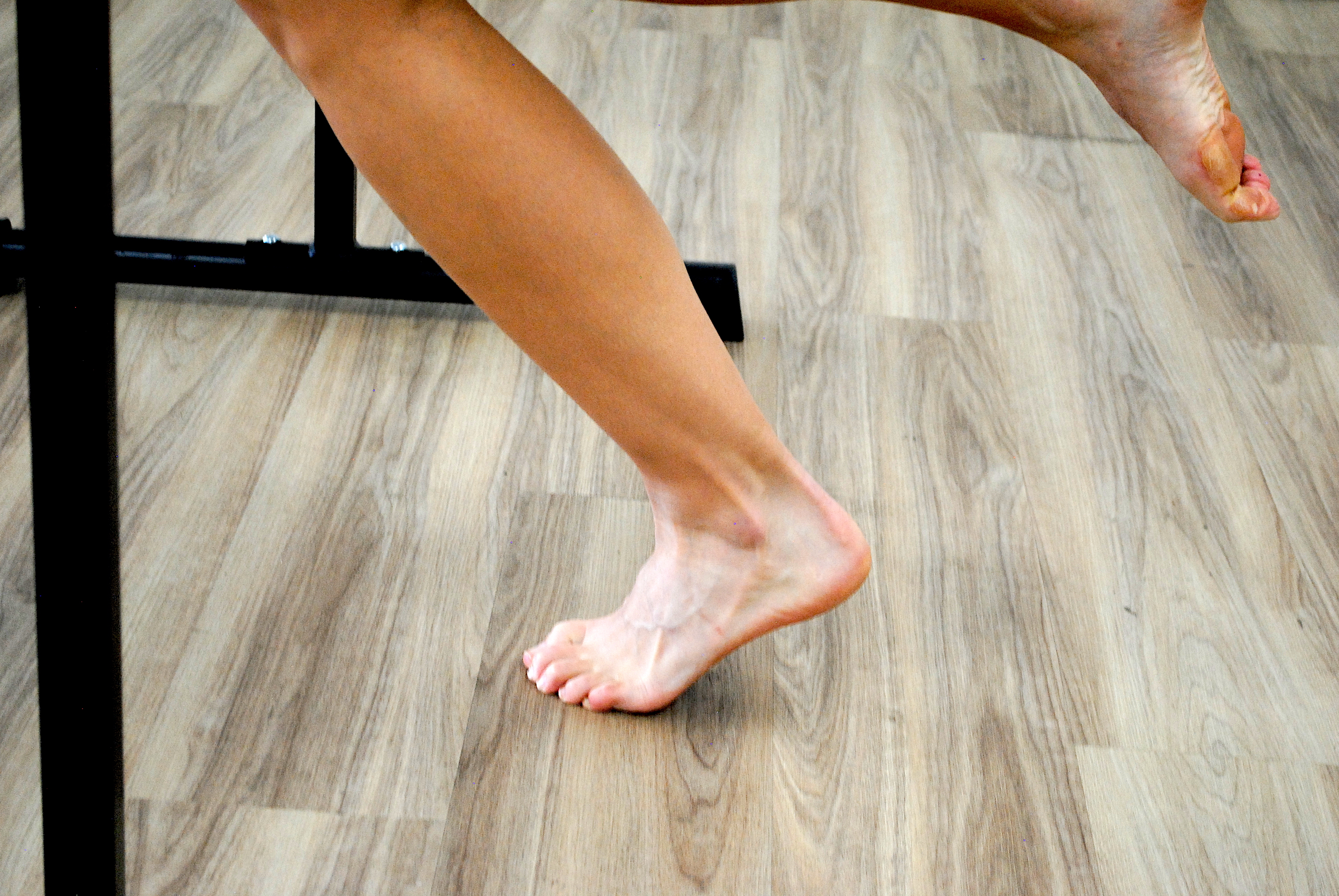
*

Table 3. *Objective testing options* *to evaluate power qualities at RTP after CMSI*

|  | **Vertical** | **Horizontal** |
| --- | --- | --- |
| Instantaneous | SL CMJ SL DJ SL concentric-only jump  SL box jump height | Single hop for distance Resisted acceleration  Resisted SL push off |
| Repeated | SL CMJ: 5-10R SL hopping: R or time  Loaded squat jumps | Forward hops in-series: 5-10R  SL bounding distance: R or time Broad jumping Resisted SL catch-ups |

SL= single leg; CMJ= countermovement jump; DJ= drop jump; R= repetitions.
